# Genome-wide association mapping reveals novel genes associated with coleoptile length in a worldwide collection of barley

**DOI:** 10.1186/s12870-020-02547-5

**Published:** 2020-07-22

**Authors:** Hao Luo, Camilla Beate Hill, Gaofeng Zhou, Xiao-Qi Zhang, Chengdao Li

**Affiliations:** 1 Western Barley Genetics Alliance, Agricultural Sciences, College of Science, Health, Engineering and Education, Murdoch University, 90 South Street, Murdoch, WA 6150 Australia; 2 Department of Primary Industries and Regional Development, Agriculture and Food, South Perth, WA Australia

**Keywords:** Coleoptile length, Deep seeding, Barley, GWAS

## Abstract

**Background:**

Drought is projected to become more frequent and severe in a changing climate, which requires deep sowing of crop seeds to reach soil moisture. Coleoptile length is a key agronomic trait in cereal crops such as barley, as long coleoptiles are linked to drought tolerance and improved seedling establishment under early water-limited growing conditions.

**Results:**

In this study, we detected large genetic variation in a panel of 328 diverse barley (*Hordeum vulgare* L.) accessions. To understand the overall genetic basis of barley coleoptile length, all accessions were germinated in the dark and phenotyped for coleoptile length after 2 weeks. The investigated barleys had significant variation for coleoptile length. We then conducted genome-wide association studies (GWASs) with more than 30,000 molecular markers and identified 8 genes and 12 intergenic loci significantly associated with coleoptile length in our barley panel. The *Squamosa promoter-binding-like protein 3* gene (*SPL3*) on chromosome 6H was identified as a major candidate gene. The missense variant on the second exon changed serine to alanine in the conserved SBP domain, which likely impacted its DNA-binding activity.

**Conclusion:**

This study provides genetic loci for seedling coleoptile length along with candidate genes for future potential incorporation in breeding programmes to enhance early vigour and yield potential in water-limited environments.

## Background

Germinating seedlings of monocotyledons have a coleoptile, which is a sheath-like tissue covering the primary leaf to protect the emerging shoot as it breaks through the soil to the surface. The coleoptile stops growing after the first true leaf pushes through the pore at the tip. The coleoptile is essential for early crop establishment and its length determines the maximum depth at which the seed can be sown [36, 40, 45, 46, 57]. If seeds are sown at a depth greater than their coleoptile length, it may result in lower emergence rate, reduced early growth, fewer tiller numbers, and decreased grain yield [17, 45, 50]. In agricultural growing areas prone to drought, the topsoil moisture can be insufficient for seed germination, and seeds need to be sown deeper to access enough moisture [31, 51] and lower temperatures [33]. Therefore, the varieties with longer coleoptiles are preferable in water-limited growing regions; for example, winter wheat grown in the low water supply areas of the Pacific Northwest of the United States are sown at depth of 10 to 20 cm [52]. In Australian varieties, coleoptile length promoting genes were found to increase the emergence of wheat seedlings at sowing depths of 12 cm without affecting plant height [12]. Deep seeding may also reduce the threat of damage by mice or other animals [8], and protect the seedlings from pre-emergent herbicides [38].

Auxins are a class of plant hormones that can modify plant cell walls, and are essential for coleoptile cell elongation and expansion [9]. In cereal coleoptiles, the most significant cell wall modifications induced by auxin are the decline of noncellulosic glucan content [23, 35, 48] and the degradation of 1,3:1,4-β-glucan [49], by activating exo- and endo-β-glucanases associated with cell walls [25, 26, 30]. This partial degradation resulted in the cell wall loosening, therefore increasing the cells’ extensibility. Auxin also enhances the synthesis of H^+^-ATPase level in the plasma membrane to increase the H^+^ extrusion into the apoplast to adjust to an optimum pH for cell wall enlargement [18]. Some studies suggest that the potassium channel gene *Zea mays K*^*+*^*channel 1* (*ZMK1*) is upregulated by auxin and essential for coleoptile elongation by maintaining K^+^ accumulation and turgor [41]. Some other cell wall-bound proteins were also reported to be key regulators for cell extension, such as α-expansin in rice [24] and β-expansin in wheat coleoptiles [14]. Nucleoside diphosphate (NDP) kinase genes were also reported to be involved in coleoptile elongation [39]. Although auxin is known to activate a group of genes responsible for cell expansion, the exact mechanisms underlying coleoptile elongation remain unclear.

Barley varieties show significant differences for coleoptile length. Paynter and Clarke (2010) [33] determined the coleoptile length for a total of 44 barley cultivars with different breeding origins, early growth habits (erect or prostrate) and pedigrees. In this collection the coleoptile length ranged from 38.7 mm (cultivar Morrell from Western Australia (WA)) to 92.9 mm (cultivar Doolup from WA), with an average of 70.2 mm. They concluded that coleoptile length was not associated with breeding origin and early growth habit in their barley collection. Takeda and Takahashi (1999) [58] scored 5082 barley and 1214 wheat varieties and found significant differences in deep-seeding tolerance, which related to the coleoptile length, first internode length and the seed size. Subsequent studies conducted QTL analyses for coleoptile length using several barley doubled haploid (DH) mapping populations: Takahashi et al. (2001) [57] used two different DH populations (Harrington × TR306 and Steptoe × Morex) and identified QTLs for deep-seeding tolerance, coleoptile length and first internode length on the long arm of chromosome 5H, corresponding with QTLs for abscisic acid and gibberellic acid response. Takahashi et al. (2008) [56] used another Harrington × TR306 population and mapped QTLs for coleoptile elongation on chromosomes 1H, 2H, 4H, 5H, 6H and 7H. However, only 127 markers for the Harrington × TR306 population, and 223 markers for the Steptoe × Morex population were available. As a result, detected QTLs spanned 2 to 5 cM intervals across the seven barley chromosomes, which was too low-resolution to pinpoint candidate genes. To date, no specific candidate genes for barley coleoptile length have been reported.

In this study, we performed genome-wide association mapping with more than 30,000 genetic markers to map the marker-trait associations (MTAs) for coleoptile length. We used a worldwide collection of mainly domesticated barley cultivars (a total of 328 accessions), including a large proportion of barley cultivars grown in the driest regions in the world such as Australia. The aims of this study were (i) to investigate the phenotypic variation of coleoptile length in a diverse worldwide collection of barley genotypes, (ii) to determine genomic regions associated with coleoptile length via GWAS, and (iii) to identify and characterise the most likely candidate genes underlying the MTAs.

## Results

### Coleoptile length in barley accessions

**Fig. 1:**
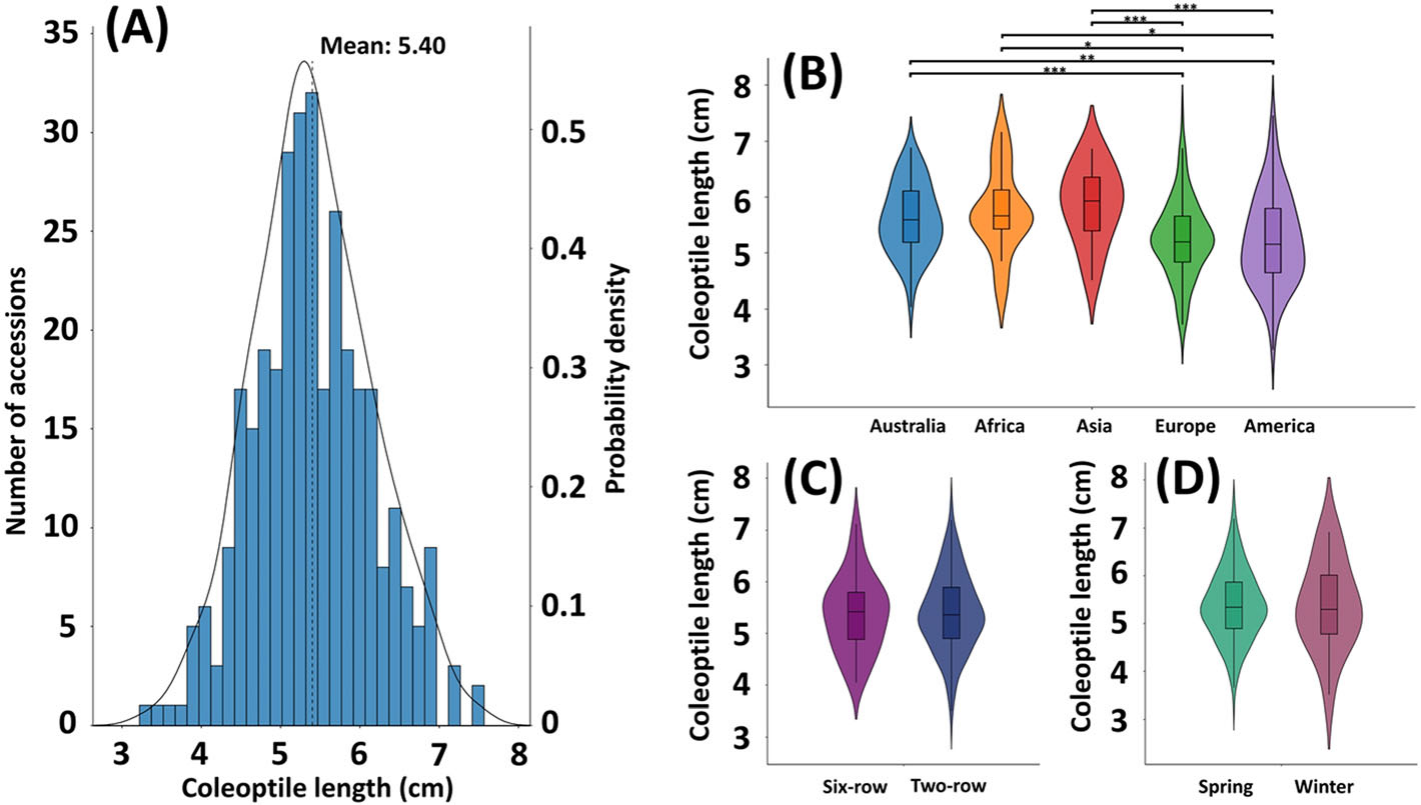
The coleoptile length in 328 barley accessions. (**a**) The distribution frequency of coleoptile length in 328 barley accessions. Comparison of coleoptile length from different origins (**b**), in different row types (**c**) and with different growth habits (**d**). The coleoptile length was averaged from the sixteen biological and three technical replicates. *: *p* < 0.05; **: *p* < 0.01; ***: *p* < 0.001

The coleoptile length recorded in two independent experiments correlated with each other significantly (R^2^ = 0.87) (all the biological replicates of coleoptile length available in Additional file 10 sheet 1) and the heritability of coleoptile length was estimated at 0.54. Therefore, the average data of the two experiments was used to represent the varieties’ coleoptile length (germinating 14 days in the dark). The investigated barleys had significant variation for the coleoptile length. The average length of the 328 barley accessions was at 5.40 cm, with the longest variety Russia24 at 7.51 cm and the shortest variety CDC Unity at 3.27 cm. The vast majority of varieties in the barley panel (292 varieties, 88.8%) had coleoptile length ranging from 4 to 6.51 cm, only 27 varieties (8.2%) ranging from 6.50 to 7.51 cm and 10 varieties (3.0%) ranging from 3.27 to 3.99 cm (Fig. 1 (a)). In this study, coleoptile above 6.50 cm is considered long, and below 4.00 cm is considered short in our dataset. The barleys from different origins, in different row types and with different growth habits were compared (Fig. 1 (b), (c) and (d)). The coleoptile length separated the barley origins into two subgroups: group 1 including Australia (mean at 5.60 cm), Africa (mean at 5.74 cm) and Asia (mean at 5.86 cm); group 2 including Europe (mean at 5.26 cm) and America (mean at 5.23 cm). The Australian varieties had longer coleoptile than European (*p* = 3.79 × 10^− 4^) and North and South American varieties (*p* = 1.26 × 10^− 3^), African longer than European (*p* = 2.51 × 10^− 2^) and North and South American (*p* = 4.26 × 10^− 2^), Asian longer than European (*p* = 1.93 × 10^− 4^) and North and South American (*p* = 8.87 × 10^− 4^) (Fig. 1 (b)). No significant difference was found among the members in either group. Barleys with different row types (two-row or six-row) had a similar coleoptile length (the two-row and six-row means were 5.40 cm and 5.37 cm, respectively) and no significant difference was detected. Similarly, no significant difference was found between the barleys with different growth habits (the spring barleys and winter barleys’ means were both 5.38 cm). In conclusion, the coleoptile length correlated to the breeding origins but not to the row types and growth habits. The varieties with relatively long coleoptile (> 6.50 cm) and their origins, row types, growth habits and coleoptile length are listed in Table S1 (Additional file 5).

### Population structure and linkage disequilibrium analysis

**Fig. 2:**
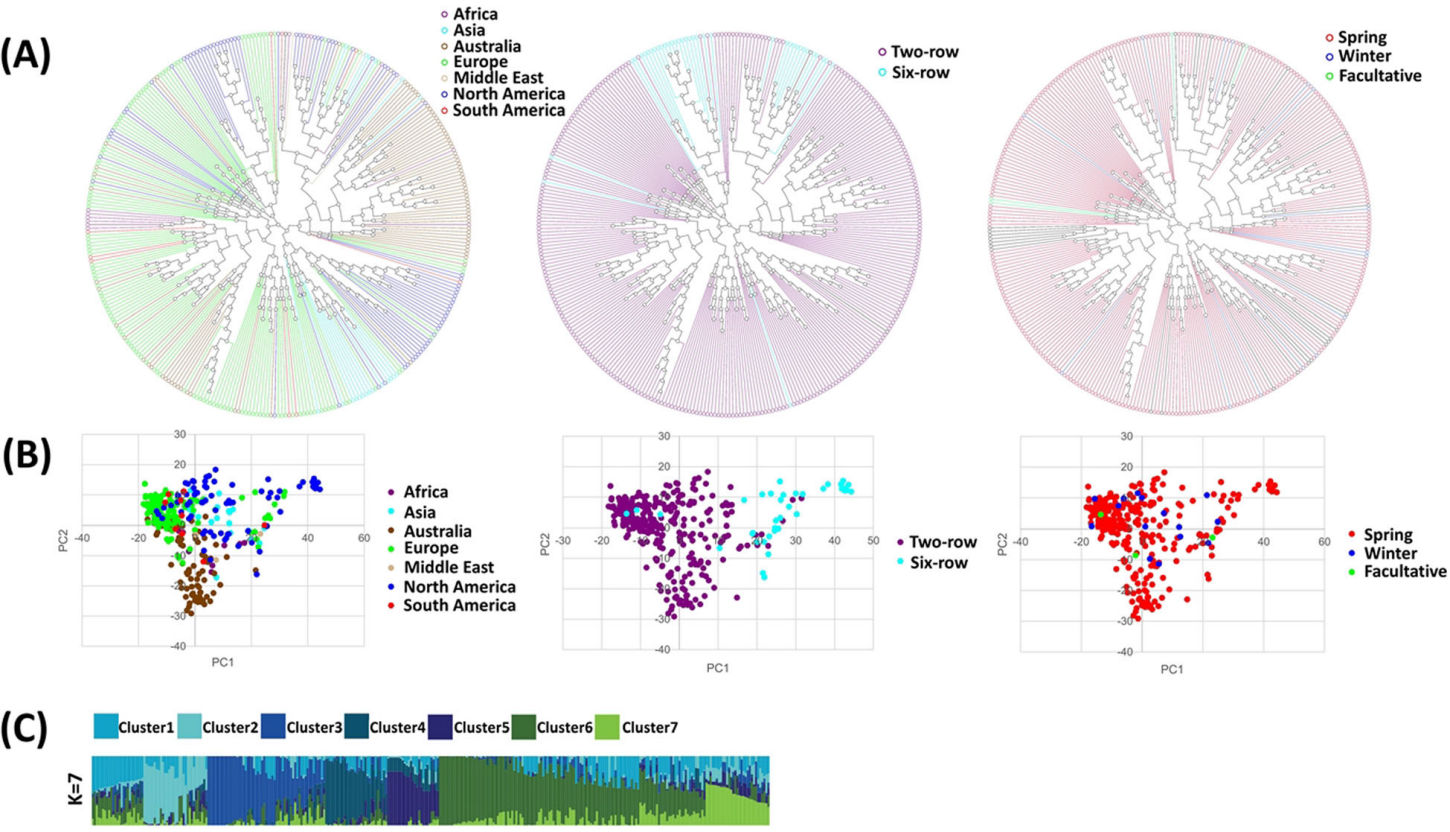
Population structure analysis of 328 barley accessions. (**a**) Phylogenetic neighbour-joining (NJ) tree of 328 barley accessions constructed based on genetic distances, highlighting their origins, row types and growth habits in different colours. (**b**) Principal component analysis (PCA) of 328 barley accessions using the first two components, according to their origins, row types and growth habits. (**c**) Population structure using ADMIXTURE for 328 worldwide barley genotypes with 19,014 SNPs. The subpopulations are presented in different colours, and the proportional membership in the population is indicated by the colour of the individual haplotypes. The CLUMPP was used to merge the membership coefficients across 100 replicate runs. According to the CV error, the K value (number of clusters) in the population of 328 accessions was determined to be 7

A neighbor-joining (NJ) tree based on genetic distances for the barley population in this study (328 barley accessions) was constructed, incorporating their origins, row types and growth habits respectively (Fig. 2 (a)). The barley collection in this study presented high genetic diversity and covered accessions from worldwide origins (35 countries), with different row types (two-row and six-row) and in different growth habits (winter, spring, and facultative) (Fig. 2 (a) (b); Figure S1 (Additional file 1)). The result of PCA with separation based on row-type, growth habit, or geographic location are presented in Fig. 2 (b).

The optimal K value (number of subpopulations) of the barley germplasm collection in this study was predicted using ADMIXTURE v.1.3.0 [1]. It showed that the optimal number of subpopulations was K = 7 according to the Δ cross-validation error (Fig. 2 (c) and Figure S2 (Additional file 2)). The population structures of 328 barley germplasm with K values from 2 to 12 were listed in Figure S3 (Additional file 3). The pairwise LD decay (r2) analysis was performed on each chromosome and decreased with physical distance (Figure S4 (Additional file 4)).

### Association analysis

The association study was performed using two subsets of markers: subset MAF01 (MAF > 0.01) including 33,146 markers and subset MAF05 (MAF > 0.05) including 23,193 markers. Significant MTAs (qFDR< 0.05) were identified on all 7 chromosomes (Figure S5 (Additional file 8) (A) and (B)). The quantile–quantile (QQ) plots indicated that the GLM model was suitable and efficient for this study (Figure S5 (Additional file 8) (C) and (D)). All the significant MTAs identified using the two marker cut-offs were listed in Table S2 (Additional file 6). Totally, there were 128 markers identified significantly associated with coleoptile length (qFDR< 0.05) using both marker subsets, representing 53 genic loci (loci within genes) and 54 intergenic loci (loci between genes) (Table S2 (Additional file 6)), explaining 4.07–6.81% phenotypic variation (r^2^). Apart from the common markers identified by both MAF01 and MAF05, MAF01 identified 49 additional markers, representing extra 19 genic loci and 9 intergenic loci (Table S2 (Additional file 6)), explaining 3.37–5.54% phenotypic variation (r^2^). Apart from the common markers identified by MAF01 and MAF05, MAF05 identified 21 additional markers, representing extra 7 genic loci and 9 intergenic loci (Table S2 (Additional file 6)), explaining 3.49–4.07% phenotypic variation (r^2^).

**Fig. 3:**
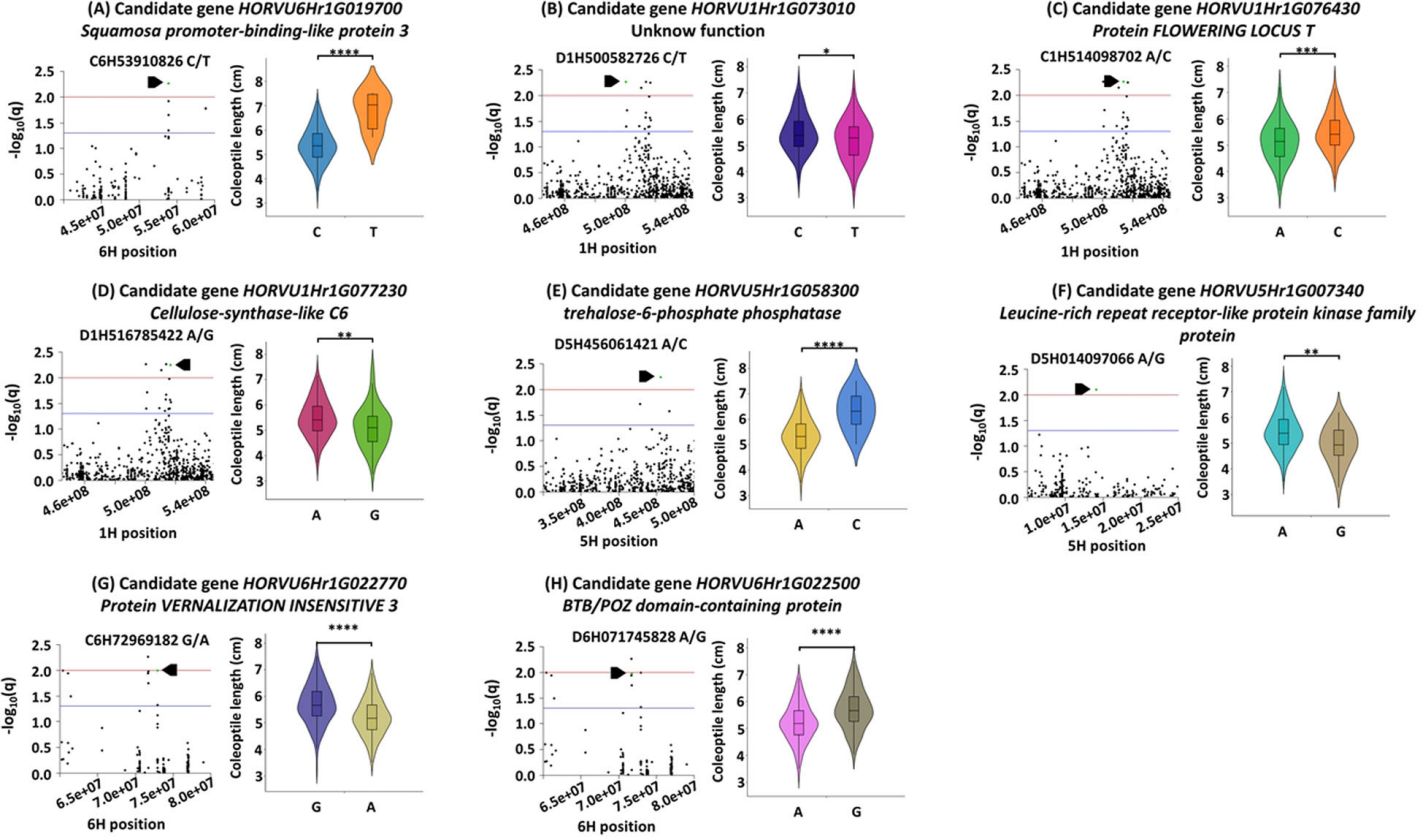
**The genomic regions of eight candidate genes had signals strongly associated with coleoptile length.** Each included the Manhattan plot showing the physical position on the chromosome (left) and the coleoptile length variation between different alleles (right). For the Manhattan plot, the q-values (−log_10_ of FDR adjusted *P*-values) were used to assess the association significance, and the signal with highest association (−log_10_(q) > 2) for each candidate gene was indicated in green colour. The threshold of MTA significance was determined by q-value cut-off at 0.05 and 0.01 and indicated by horizontal lines in different colours (0.05 (blue) and 0.01 (red)). For the coleoptile length variation plot, * p < 0.05; ** p < 0.01; *** p < 0.001; **** *p* < 0.0001. The coleoptile length was averaged from the sixteen biological and three technical replicates

The highly significant associated loci (qFDR< 0.01, −log_10_(q) > 2.0) identified by either MAF01 or MAF05 are listed in Table 1. There were 5 genic loci identified by MAF01 and MAF05 in common, including *HORVU1Hr1G073010* (unknown function), *HORVU1Hr1G076430* (*Protein FLOWERING LOCUS T* (*FT*)), *HORVU1Hr1G077230* (*Cellulose-synthase-like C6* (*CSLC6*)), *HORVU5Hr1G058300* (*Trehalose-6-phosphate phosphatase* (*TPPB*)) and *HORVU5Hr1G007340* (*Leucine-rich repeat receptor-like protein kinase family* (*LRR-RLK*)). One genic locus within gene *HORVU6Hr1G019700* (*Squamosa promoter-binding-like protein 3* (*SPL3*)) was only detected in MAF01. Two genes harbouring genic loci, *HORVU6Hr1G022770* (*Protein VERNALIZATION INSENSITIVE 3* (*VIN3*)), and *HORVU6Hr1G022500* (*BTB/POZ domain-containing protein* (*BTBD*)) were only detected in MAF05. The top significantly associated markers (with the lowest qFDR) for each candidate gene are summarized for their effect on the coleoptile length (Fig. 3 and Additional file 10 sheet 4). In conclusion, all the associated markers within genes had strong effects on the coleoptile length. There were 12 intergenic loci identified by MAF01, MAF05 or both. Possible genes responsible for coleoptile length were searched around the loci and listed in Table S3 (Additional file 7).

**Table 1:** Loci significantly associated with coleoptile length using two MAF (q < 0.01)

GLM model MAF > 0.05						
SNP ID^*^	Alleles	MAF†	R^**2**^ (%)^**§**^	q value^**¶**^	Candidate gene ID^**δ**^	Annotation
1H500582726	C:T	0.24	6.52	8.75E-03	*HORVU1Hr1G073010*	unknown function
1H514098702	A:C	0.23	6.68	8.75E-03	*HORVU1Hr1G076430*	*Protein FLOWERING LOCUS T*
1H516785422	A:G	0.2	6.28	8.75E-03	*HORVU1Hr1G077230*	*Cellulose-synthase-like C6*
2H026308852	A:G	0.25	6.32	8.75E-03	.^ζ^	.
2H640651652	G:C	0.14	6.45	8.75E-03	.	.
4H015498974	C:G	0.09	6.33	8.75E-03	.	.
5H456061421	A:C	0.06	6.45	8.75E-03	*HORVU5Hr1G058300*	*trehalose-6-phosphate phosphatase*
6H071685909	C:T	0.35	6.65	8.75E-03	.	.
6H114729800	T:C	0.32	6.81	8.75E-03	.	.
2H025712787	C:T	0.44	6.14	8.87E-03	.	.
3H159754241	A:G	0.11	6.16	8.87E-03	.	.
5H014097066	A:G	0.08	6.10	8.87E-03	*HORVU5Hr1G007340*	*Leucine-rich repeat receptor-like protein kinase family protein*
6H095840955	T:C	0.12	6.12	8.87E-03	.	.
1H510799369	A:C	0.26	6.01	9.48E-03	.	.
3H325190801	T:G	0.05	6.00	9.48E-03	.	.
4H009849212	C:T	0.23	5.91	9.48E-03	.	.
6H060392776	T:C	0.36	5.92	9.48E-03	.	.
6H72969182	G:A	0.37	5.91	9.48E-03	*HORVU6Hr1G022770*	*Protein VERNALIZATION INSENSITIVE 3*
6H071745828	A:G	0.36	5.87	9.77E-03	*HORVU6Hr1G022500*	*BTB/POZ domain-containing protein*
**GLM model MAF > 0.01**						
**SNP ID**	**Alleles**	**MAF**	**R**^**2**^**(%)**	**q value**	**Candidate gene ID**	**Annotation**
1H514098702	A:C	0.23	6.71	5.45E-03	*HORVU1Hr1G076430*	*Protein FLOWERING LOCUS T*
4H015498974	C:G	0.09	6.42	5.45E-03	.	.
6H53910826	C:T	0.02	5.54	5.45E-03	*HORVU6Hr1G019700*	*Squamosa promoter-binding-like protein 3*
6H53910924	T:A	0.02	5.54	5.45E-03	*HORVU6Hr1G019700*	*Squamosa promoter-binding-like protein 3*
6H53911180	T:A	0.02	5.54	5.45E-03	*HORVU6Hr1G019700*	*Squamosa promoter-binding-like protein 3*
6H53911713	A:C	0.02	5.54	5.45E-03	*HORVU6Hr1G019700*	*Squamosa promoter-binding-like protein 3*
6H53912147	C:T	0.02	5.54	5.45E-03	*HORVU6Hr1G019700*	*Squamosa promoter-binding-like protein 3*
6H53912695	T:C	0.02	5.54	5.45E-03	*HORVU6Hr1G019700*	*Squamosa promoter-binding-like protein 3*
6H53913050	A:C	0.02	5.54	5.45E-03	*HORVU6Hr1G019700*	*Squamosa promoter-binding-like protein 3*
6H53913075	C:T	0.02	5.54	5.45E-03	*HORVU6Hr1G019700*	*Squamosa promoter-binding-like protein 3*
6H53913335	G:A	0.02	5.54	5.45E-03	*HORVU6Hr1G019700*	*Squamosa promoter-binding-like protein 3*
6H53913549	C:T	0.02	5.54	5.45E-03	*HORVU6Hr1G019700*	*Squamosa promoter-binding-like protein 3*
6H53915124	T:A	0.02	5.54	5.45E-03	*HORVU6Hr1G019700*	*Squamosa promoter-binding-like protein 3*
6H071685909	C:T	0.35	6.53	5.45E-03	.	.
6H114729800	T:C	0.32	6.69	5.45E-03	.	.
1H500582726	C:T	0.24	6.37	5.50E-03	*HORVU1Hr1G073010*	*unknown function*
2H640651652	G:C	0.14	6.35	5.50E-03	.	.
1H516785422	A:G	0.2	6.29	5.63E-03	*HORVU1Hr1G077230*	*Cellulose-synthase-like C6*
2H026308852	A:G	0.25	6.29	5.63E-03	.	.
5H456061421	A:C	0.06	6.25	5.81E-03	*HORVU5Hr1G058300*	*trehalose-6-phosphate phosphatase*
6H095840955	T:C	0.12	6.22	5.82E-03	.	.
2H025712787	C:T	0.44	6.20	5.83E-03	.	.
1H510799369	A:C	0.26	6.08	7.09E-03	.	.
3H159754241	A:G	0.11	6.03	7.67E-03	.	.
5H014097066	A:G	0.08	5.99	7.98E-03	*HORVU5Hr1G007340*	*Leucine-rich repeat receptor-like protein kinase family protein*
3H325190801	T:G	0.05	5.90	9.17E-03	.	.

*the ID consists of the chromosome number followed by the marker’s physical position

†minor allele frequency

^§^contribution to phenotypic variation

^¶^adjusted *P* value, significant at q < 0.01; the association list of q < 0.05 see supplementary data

^δ^annotated in barley genome assembly IBSC v

^ζ^intergenic region

### Major candidate gene for coleoptile length

The markers within gene *SPL3* showed association with coleoptile length for both MAF01 and MAF05. In MAF01, there were 5 markers on *SPL3* identified to be significant (0.01 < qFDR< 0.05) and 11 markers on *SPL3* showed highest significance of all associations (qFDR = 5.4 × 10^− 3^). In MAF05 there were 2 markers on *SPL3* identified to be significant (0.01 < qFDR< 0.05) (Table S2 (Additional file 6)). Although the significant markers within eight candidate genes all had strong effects on the coleoptile length, the marker C6H53910826 (and other 10 markers with qFDR = 5.4 × 10^− 3^ in MAF01) on *SPL3* showed the most significant effect: the mean length was at 5.37 cm when the allele C presented and 6.79 cm when the allele T presented (*p* = 2.85 × 10^− 6^) (Fig. 3 (a)). In conclusion, multiple markers on *SPL3* had been identified to be significantly associated by different methods and some of the markers had the highest association index (qFDR = 5.4 × 10^− 3^) and had the strongest effect on the phenotype. Therefore, *SPL3* was considered the major candidate gene associated to the coleoptile length in this study.

**Fig. 4:**
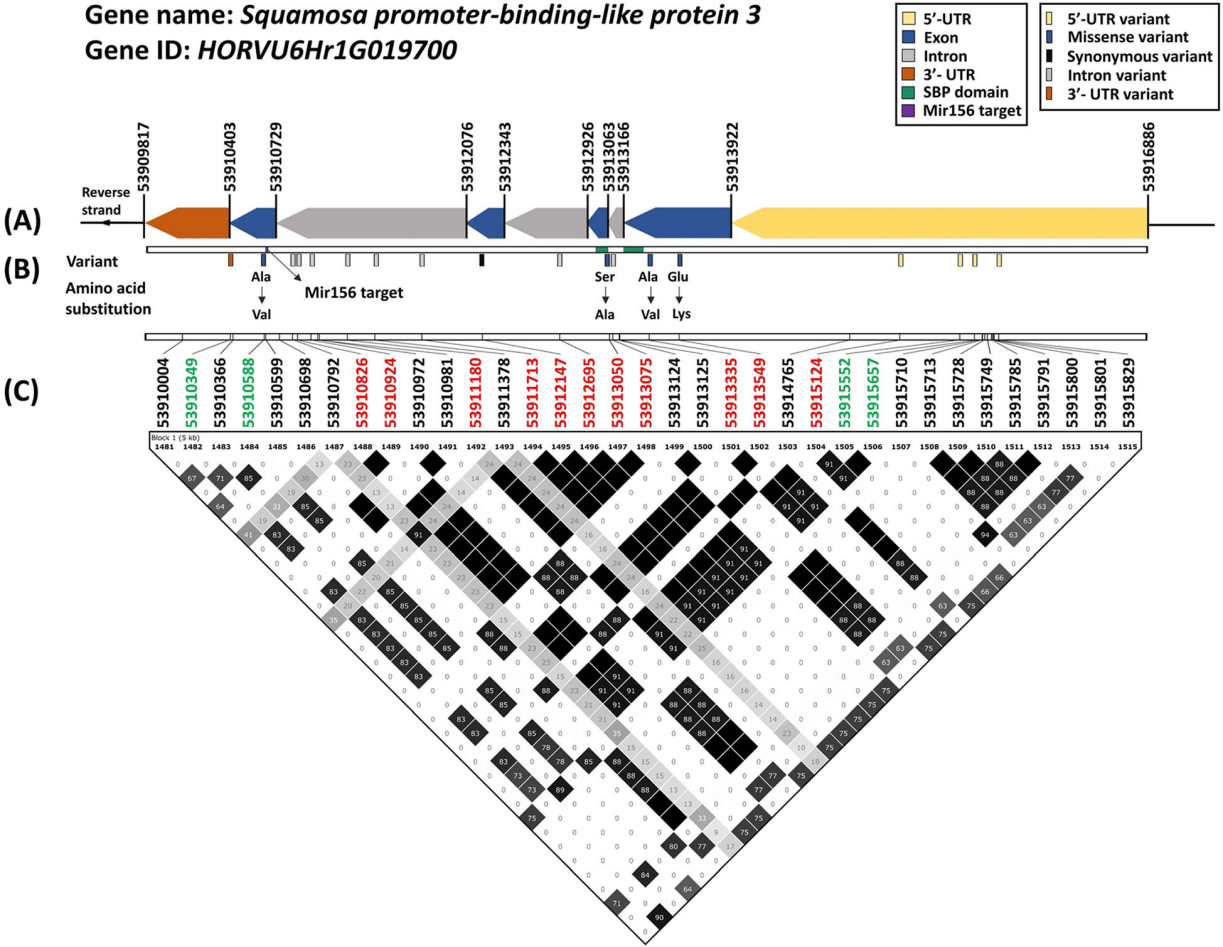
Summary of the *SPL3* gene structure, amino acid substitution and the local LD haplotype blocks. **a** The gene structure was annotated in barley genome assembly IBSC v2. **b** the amino acid substitutions were predicted by detected GWAS markers. (**c**) The LD plot (made by Haploview) showed the r^2^ values between pairs of SNPs (× 100); white colour indicated *r*^*2*^ = 0; grey colour indicated 0 < *r*^*2*^ < 1; black colour indicated *r*^*2*^ = 1. The four-gamete rule method was used to compute the haplotype block in the *SPL3* genomic region. The green font showed the SNPs with significant association (0.01 < q value< 0.05) in the GLM model. The red font showed the SNPs with highly significant association (q value< 0.01) in the GLM model

*SPL3* is a transcription factor gene located on chromosome 6H:53,909,817 to 53,916,886 (7070 bp), consisting of 5’UTR, 3’UTR, four exons and three introns (Fig. 4 (a)). The gene encodes a protein with 474 amino acids (aas). The conserved SBP domain is central functional region of this transcription factor, and contains a plant-specific DNA-binding domain. A miR156 binding target was also identified on the last exon of *SPL3*. All the variants and amino acid substitutions are summarized in Fig. 4 (b). There were five variants on exons, including four missense variants and one synonymous variant. The missense variant at position 53,913,050 replaced serine with alanine in the SBP domain, likely impacting its DNA-binding activity. Furthermore, this marker C6H53913050 was one of the markers showing highest significance in this study (qFDR = 5.4 × 10^− 3^). Three other missense variants included glutamic acid replaced with lysine at position 53,913,549, alanine replaced with valine at position 53,913,335 and 53,910,588. However, no variant was found on the miR156 binding target (Fig. 4 (b)). Five variants were found in 5′ or 3′ UTR and eight variants were in the introns (Fig. 4 (B)), which potentially affected gene expression. Fig. 4 (c) showed the position of all detected variants, including significant association and non-significant association with coleoptile length, and the LD plot surrounding these markers.

**Fig. 5:**
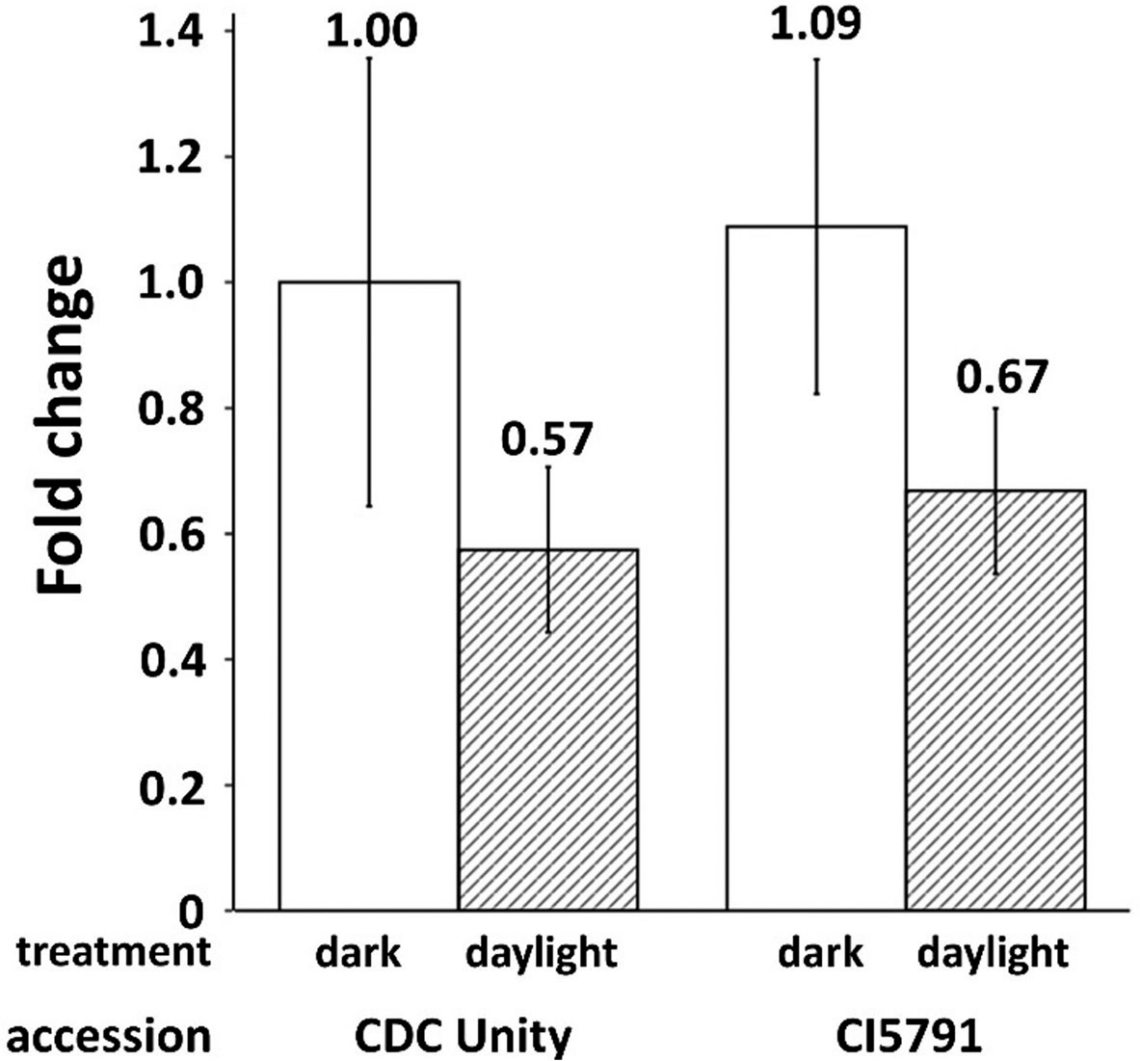
The relative expression (fold change) of *SPL3* gene in coleoptile of CDC Unity and CI5791. The ‘dark’ indicated the gene expression in etiolated coleoptile tissue and the ‘daylight’ indicated the gene expression in normal coleoptile tissue under natural light. The expression was normalized using reference gene (actin) and relative to the etiolated coleoptile tissue of CDC Unity (converted to 1)

To further understand the role of *SPL3* gene in barley coleoptile growth, we measured its expression in coleoptile tissue of two varieties (CDC Unity (coleoptile length 3.27 cm) and CI5791 (coleoptile length 7.17 cm)), representing two major haplotypes of *SPL3* (C/T at marker C6H53910826) in the population. The comparison of the *SPL3* expression between CDC Unity and CI5791, and in dark and under natural daylight is presented in Fig. 5 (the raw data available in Additional file 10 sheet 3). The data represented the actin-normalized target gene expression relative to control (the *SPL3* gene expression in coleoptile of CDC Unity in dark) (considered as 1). There was no statistically significant difference between CDC Unity and CI5791, either in dark or under natural daylight. However, in CDC Unity, the expression under light decreased by 43% compared with dark conditions. Similarly, in CI5791, the expression under light decreased by 39% compared with dark conditions. In conclusion, the coleoptile length variation between two *SPL3* alleles was not due to the gene expression, but for the both alleles there was a steady decline in gene expression when the coleoptile was exposed under daylight.

## Discussion

More frequently occurring drought conditions as result of changing climate has become a major challenge in many growing regions of the world. Long coleoptile barleys have the potential to help mitigate limitations on emergence and improve the field establishment of barleys in water limited areas. In this study we performed GWAS on coleoptile length using 328 barley accessions with more than 30,000 molecular markers. As a result, the coleoptile length showed significant variation in this barley population and the GWAS identified 8 genes and 12 intergenic loci with high significance, providing a valuable source for selecting long coleoptile barleys in the future (coleoptile above 6.50 cm is considered long, and below 4.00 cm is considered short in our dataset).

In this study, the seeds were sown in washed river sand, a commonly used method for coleoptile measurement [44, 56], and more closely related to the seeds’ natural germination conditions compared with the wet filter paper method [6]. Previous studies indicated that, apart from the genetic factors, both the seed source (seed age, storage condition, growing location, seed size and seed viability) and the germination methodology may affect the coleoptile length [42, 56], which explained the variations of our measurement compared to earlier studies [6, 40].

Allele frequency is a key factor affecting the power of GWAS and impacting on detecting marker-trait associations [2]. To date, most GWAS studies focused solely on common variants (MAF ≥ 0.05). However, there are reported cases in barley where rare alleles accounted for natural allelic variation in certain individuals which are of importance for crop improvement and further genetic studies [62]. To also discover highly informative rare alleles, we included two MAF cut-offs in this study – one for common alleles set at ≥5%, and one for rare alleles set at ≥1%.

*SPL3* is the major candidate gene for coleoptile length in this study. The SPL3 protein belongs to SBP family and contains the conserved SBP domain. It is an important plant-specific transcription factor regulating plant growth and development, and the two zinc finger structures are located in the core region of the SBP domain [32]. According to the gene variants, there are four amino acid substitutions, one of which is a serine to alanine change located in the second zinc-finger structure of SBP domain [10]. Although the substitution is not on the most highly conserved part of the zinc-finger structure (Figure S6 (Additional file 9)), it potentially affects the SPL3 DNA binding affinity or protein interaction. The miR156 binding region identified on *SPL3* was a key target for post-transcriptional regulation by microRNA [10], but no variant was detected on it. We showed that *SPL3* is expressed in the coleoptile tissue and the expression increased in dark conditions, in accordance with the light sensitivity of coleoptile growth. Although the expression in dark only increased by 60–70% compared with daylight conditions, the effect can be significantly amplified since SPL3 is a transcription factor potentially activating other genes [13, 60]. However, the gene expression between two haplotypes in the population was identical, so the coleoptile length variation of the two genotypes was possibly due to the protein difference rather than the expression. However, the gene expression was only tested at one point of time (7 days) during germination, which possibly missed the window of statistically significant differences in expression between the two varieties. A time course over the germination process can be done to draw a more conclusive result on the expression difference. Furthermore, other factors beyond protein sequence variation may also influence a protein’s function, such as post translational modification and interaction with other components [11, 15]. At this stage, how *SPL3* variants affect the coleoptile length between two varieties remains unclear. Although in our initial GWAS we provide statistical evidence that a gene is likely to harbour a causal variant, as in any GWAS study additional statistical methods and follow-up functional studies are needed to discriminate likely functional variants from variants that in LD with the functional variants. Further studies are also needed to determine the function and the downstream target of this gene, as well as how it affects coleoptile growth in barley.

A number of candidate genes identified in this study were shown to regulate cell growth in other plants. *CSLC6* on 1H, encoding an enzyme involved in cell wall biosynthesis, was determined a candidate gene due to highly significant associations with coleoptile length. The gene, a member of the cellulose-synthase-like family, was proposed to encode the catalytic subunit of enzymes that synthesize hemicellulose backbones [47]. A previous study showed that hemicelluloses were involved in regulation of wall modification, elongation and growth [61]. Therefore, this gene may influence the coleoptile length by regulating cell wall synthesis. *GA2ox* (*HORVU1Hr1G076730*) on 1H was identified as another candidate gene, and it is also a known semi-dwarf gene [34]. This gene is essential in the Gibberellin (GA) catabolic pathway and regulates plant growth by inactivating endogenous bioactive GAs. Previous studies indicated that many semi-dwarf genes had a negative effect on coleoptile length [5, 16]. This gene may regulate the plant growth by GA pathway in multiple aspects, including the coleoptile elongation. *EXPB2* (*HORVU1Hr1G054230*) on 1H was only identified by MAF01 with significant association. Members in this gene family had been showed to regulate coleoptile growth in other cereal crops [14, 24]. Previous studies showed that the wheat coleoptile length was controlled by multiple genes on different chromosomes, and the combination of QTLs with minor effect could improve coleoptile length obviously [37, 46]. Further work needs to be done to investigate how the genes and loci identified in this study had an impact on barley coleoptile and it would provide an opportunity to breed barley varieties with preferable coleoptile length by manipulating multiple genes/loci.

For cereal crops grown in drought prone regions, cultivars with longer coleoptile can emerge quicker and have better early vigour [3]. Longer coleoptiles can not only protect the crops from early drought but also improve water use efficiency, and are more sustainable in low water supply areas [29]. In the past decades, coleoptile elongation has been studied mainly in wheat, with a main focus on dwarf or semi-dwarf genes that had only little effect on the coleoptile length [16]. Only a few other studies announced coleoptile length-related loci other than dwarf genes [5]. In this study, by genome wide association analysis, 20 loci were identified at high significance (Table 1) and more than 100 loci were detected at lower significance (Table S2 (Additional file 6)), providing a wide range of genetic factors interacting with coleoptile elongation. This study provided a comprehensive overview of the genetic pool regulating coleoptile length. The genes detected in this study need further investigation regarding to their variation in the barley accessions and their mechanism regulating coleoptile cell growth. It opens the opportunity to understand the genetic network of crop early vigour and field establishment.

## Conclusions

In this study, a worldwide collection of barleys (328 accessions) showed a large phenotypic variation for coleoptile length. The genome wide association analysis using more than 30,000 markers identified 8 genes and 12 intergenic loci with high significance (qFDR< 0.01), as well as 71 genes and 60 intergenic loci with lower significance (0.01 < qFDR< 0.05). *SPL3* was determined as the novel major candidate gene for coleoptile length in this study. It had the strongest effect on the coleoptile length among the candidate genes and was identified by multiple markers and different methods. *SPL3* was expressed in the coleoptile but no significant expression difference was found between haplotypes. The substitution of serine for alanine in the second zinc-finger structure of SBP domain in *SPL3* likely impacted its DNA-binding activity. This work provides a valuable overview of genetic factors responsible for coleoptile length in barley, detecting genome-wide loci with improved resolution, making it easier to pinpoint the responsible genes in the future research. It provides opportunity to improve seedling early vigour and stand establishment for barley and other crops by better understanding the mechanism regulating coleoptile growth.

## Methods

### Plant material

In this study, 328 barley accessions were selected from a barley diversity panel described previously [20, 22]. The selection included cultivated, landrace, and research barley accessions representing the diversity on coleoptile length, geographic origin (35 countries in Europe, North and South America, Africa, Asia and Australia) and cultivated barley lifeforms (two-row and six-row genotypes; winter, spring, and facultative growth habits). All plant materials used in this study are provided in Figure S1 (Additional file 1) and Additional file 10 sheet 1.

### Phenotyping

Seeds with normal size and plumpness were selected from each accession of 328 varieties. The commercially available washed river sand was sourced from the Avon river (Perth, Western Australia), and was tested for its properties by the CSBP Soil and Plant Analysis Laboratory (Perth, Western Australia). It contained no chemical residues that may affect plant growth including coleoptile growth. Seeds of each barley genotype were sown at 2 cm depth in individual 8x8cm cells of germination trays filled with washed river sand (12% v/w moisture) and stored in the dark at 20 °C. After 14 days the etiolated seedlings were pulled out from the river sand and the coleoptile length from the embryo end of grain to the coleoptile tip was measured manually using a ruler. Eight seeds (eight biological replicates) from each accession were used for coleoptile length measurement, and each seed was measured for three times (three technical replicates). The experiment was repeated once. Absolute measurements that deviated from mean values by more than 2 standard deviation units were considered as outliers and set as missing values. The average values of the sixteen biological replicates were used to represent the varieties’ coleoptile lengths.

### DNA extraction and high-throughput genotyping

The collection of 328 barley varieties were grown in a naturally lit glasshouse at Murdoch University, Perth, Western Australia (32°04′S, 115°50′E) and harvested at the three-leaf stage (three plants per each variety). Genomic DNA extraction was performed using a rapid cetyl-trimethyl- ammonium bromide (CTAB) method [53].

We used a combination of three sequencing methods (target-enrichment sequencing, low-coverage whole genome sequencing (WGS), and DArTseq) to capture variation in and around the gene-containing regions of the 328 barley genotypes. The target-enrichment sequencing of genomic DNA regions and low-coverage whole genome sequencing were performed as described before [20, 22]. Genotyping-by-sequencing (GBS) by DArTseq was performed using the DArTseq platform as detailed previously [21]. Stringent filtering steps were adopted to obtain clean data. All genotype data were combined, filtered based on duplicates and MAF > 1% or MAF > 5%, and imputed using BEAGLE v.4.1.

### Population structure and genotypic data analysis

Genetic distance based on IBS (identity-by-state similarity) were calculated and a Neighbor-Joining (NJ) dendrogram was computed, all using TASSEL v.5.2.39 software [7].

The model-based clustering algorithm of ADMIXTURE v.1.3.0 was used to investigate subpopulation structure of the barley diversity panel. Prior to population structure analysis in ADMIXTURE, the genotype dataset was LD pruned using Plink v1.939 with the window size set to 50 kb, step size to 5, and the pairwise r^2^ threshold set to 0.5, yielding 19,014 genetic variants. The K value was set from 1 to 20 to do a preliminary analysis with 100 replicate runs and a 10-fold cross validation (CV) procedure was performed as described before [20, 21]. ADMIXTURE’s CV error values were used to select the most likely K-value. The optimization of the replicate alignments for each K-value was performed using CLUMPP [27]. The plots were made using optimal K value by Pophelper v.2.2.3 (http://royfrancis.github.io/pophelper/) implemented in R software (http://www.R-project.org/) as detailed previously [21].

Principal component analysis (PCA) was also conducted based on all markers data using TASSEL v.5.2.39 to summarize the genetic structure and variation present in the barley germplasm. The first two principal components were plotted against each other using ‘scatter plot’ function in Microsoft Excel 2016. NJ trees were constructed using the Java application Archaeopteryx v.0.9909 [19] based on genetic distances calculated in TASSEL v.5.2.39.

### LD analysis

Genome-wide LD analysis was performed by pair wise comparisons among the intra-chromosomal genetic markers using Plink v.1.93 [43]. LD was estimated by using squared allele frequency correlations (r^2^) between the intra-chromosomal pairs of loci. To investigate the extent of LD decay, intra-chromosomal r^2^ values were plotted against the physical distance (kb) between markers. Curves were fitted by second-degree LOESS using R software v.3.5.1 (http://www.R-project.org/).

### Association analysis

Genome wide association studies were performed using 33,146 (MAF > 0.01) and 23,193 (MAF > 0.05) genetic markers, respectively in TASSEL [7].

*P*-values for putative MTAs, population structure (Q) and the kinship matrices (K) were computed by many different statistical models to prevent spurious associations. The following models were tested: i) Naïve model: GLM without any correction for population structure; ii) P-model: GLM with Q (3, 5, and 7 PCs) or K matrix (matrix of genetic similarity based on simple SNP matching coefficients, which was also used for constructing the neighbour-joining tree) as a correction for population structure; iii) Q + K model: Compressed Mixed Linear Model (MLM) with population structure (Q) matrix (PCs: 3, 5, 7) and K matrix (matrix of genetic similarity based on simple SNP matching coefficients, which was also used for constructing the neighbour-joining tree) as a correction for population structure. Results were compared, and according to the quantile-quantile (Q-Q) plot, for this study the GLM with population structure (Q) matrix (the first three PCs) as a correction for population structure was most suitable out of all the tested models.

For the GLM model and analysis, the qvalue was calculated in different testing using R package (R 3.4.2) as described previously [20, 54, 55], to assess the significance of MTAs. Lambda was selected as 0 which estimates πi(0) = 1, which produces a list of significant tests equivalent to the [4] procedure and is considered a conservative case of the qvalue methodology. Only markers with qFDR < 0.05 were considered to be significant. The Manhattan plot was drawn with qqmanv.0.1.4 [59].

Broad-sense heritability (H^2^) was calculated in R statistical software using the ‘lme4’ package and the standard ‘anova’ function according to the following equation by treating genotype and environment as random effects:

1 $$ {\mathrm{H}}^2=\frac{\upsigma_{\mathrm{a}}^2}{\upsigma_{\mathrm{a}}^2+{\upsigma}_{\mathrm{e}}^2} $$

where ${\sigma}_a^2$ and ${\sigma}_e^2$ represent the variance derived from genotypic and environmental effects, respectively.

### Candidate gene expression

Seeds with normal size and plumpness were selected from barley varieties CDC Unity (short coleoptile haplotype) and CI5791 (long coleoptile haplotype), representing the two major haplotypes of the primary candidate gene *Squamosa promoter-binding-like protein 3* (*SPL3*) in the population (The single-nucleotide variants between short and long coleoptile haplotypes available in Additional file 10 sheet 2; the amino acid change between short and long coleoptile haplotypes available in Fig. 4 (b) with short haplotype at the top and long haplotype at the bottom). Eight seeds per variety were sown at 2 cm depth in washed river sand (12% v/w moisture) and stored at 20 °C in the dark or under normal daylight. The coleoptiles of eight seedlings were collected together as one biological replicate. Three biological replicates and three technical replicates were used for the gene expression assay. The coleoptile was collected at day 7 and ground to a fine powder with liquid nitrogen. The total RNA was extracted using TRIsure (Bioline, #38032) following the manufacturer instruction. The RNA extract was quantified using NanoDrop™ One Microvolume UV-Vis spectrophotometer (Thermo Scientific) and approximately equal amount of RNA (~ 1 μg) was used for reverse transcription. The reverse transcription was done using SensiFAST™ cDNA Synthesis Kit (Bioline, # 65053) as per manufacturer instruction. The real-time PCR was performed using SensiFAST™ SYBR® Lo-ROX Kit (Bioline, #94005) and ViiA™ 7 Real-Time PCR System (Applied Biosystems), performed in triplicate for each cDNA sample. A pair of primers were designed to bind uniquely to the *SPL3* gene and they were made straddle the intron such that they anneal only to the cDNA synthesized from RNA to avoid DNA contamination: SPL3_exp_F (5′-ACAGTGCAGCCGGTTTCATG-3′) and SPL3_exp_R (5′-GAACATATGGAGCCTGACCGA-3′) (product size 197 bp; annealing temperature 62 °C). The target gene expression was normalized using Actin as a reference [28]. The thermocycling was set as follows: 96 °C for 2 min, 40 cycles at 96 °C for 15 s, 62 °C for 15 s and 72 °C for 15 s, and a final extension at 72 °C for 5 min. The melt-curve analysis was performed according to the following protocol: 95 °C for 15 s (ramping rate 1.6 °C/s), 60 °C for 1 min (ramping rate 1.6 °C/s) and 95 °C for 15 s (ramping rate 0.05 °C/s). The relative expression levels of target genes were determined as 2^−△Ct^. The size of the PCR product was estimated by 2% agarose gel.

## Supplementary information

**Additional file 1 Figure S1.** Diversity of barley varieties. a) The geographic distribution of barley accessions. Numbers above the bars showed the varieties numbers from each geographical origin. b) Row type and c) growth habit in barley accessions. Numbers on the top of the bars showed the varieties numbers in different row-types and life forms.

**Additional file 2 Figure S2.** Selection of the optimal number of K (genetic subpopulations). The red line indicated the optimal K value (the most likely number of subpopulations) as 7 based on Δ cross-validation error and standard error in barley accessions.

**Additional file 3 Figure S3.** Population structure analysis using ADMIXTURE for 328 worldwide barley genotypes with 19,014 SNPs. Different populations are showed in different colours. The proportional membership in the population is indicated by the colour of the individual haplotypes. CLUMPP was used to merge the membership coefficients for each population with 100 replicate runs. The number of clusters (K) in 328 barley varieties was determined to be 7 based on the CV error.

**Additional file 4 Figure S4:** The LD decay of marker pairs in the worldwide collection of domesticated barley varieties. X-axis indicated the distance (kbp) and Y-axis indicated the mean r^2^ values of all intra-chromosomal pairs of SNPs. The red lines are the LOESS fitting curves fit by second-degree loess.

**Additional file 5 Table S1** Barley accessions with long coleoptile.

**Additional file 6 Table S2** Loci significantly associated with coleoptile length using two MAF (q < 0.05)

**Additional file 7 Table S3** Intergenic Loci with highly significant association (qFDR < 0.01) and candidate genes

**Additional file 8 Figre S5**. Manhattan plots and quantile–quantile (QQ) plot of coleoptile length. (A) The Manhattan plot of coleoptile length by GLM model using minor allele frequency (MAF) < 0.05. (B) The Manhattan plot of coleoptile length by GLM model using minor allele frequency (MAF) < 0.01. The significance of marker-trait associations was presented using q values (−log10 of FDR adjusted *p*-values). The blue dashed lines indicated the significant threshold at qFDR< 0.05 and the red dashed lines indicated the significant threshold at qFDR< 0.01. (C) QQ plot for coleoptile length using q values (−log10 of FDR adjusted p-values) by GLM model with MAF < 0.05. (D) QQ plot for coleoptile length using q values by GLM model with MAF < 0.01.

**Additional file 9 Figure S6**. The amino acid substitution site and the conserved motifs on SBP domain of gene *SPL3*. Zn-1: zinc finger 1; Zn-2: zinc finger 2; NLS: nuclear localization signal. Blue colour indicated the most conserved region on zinc finger 1. Red colour indicated the most conserved region on zinc finger 2. Purple colour indicated the most conserved region on nuclear localization signal.

**Additional file 10 **The dataset supporting the conclusions of this article. Sheet 1: The coleoptile length data including experiments conducted across two years (2017 and 2019) with a total of 16 biological replicates. The length unit is centimetre. Missing data due to germination failure is left blank; sheet 2: The single-nucleotide variants between short and long coleoptile haplotypes. 0 represented the reference allele and 1 represented the alternative allele; sheet 3: The raw data for *SPL3* gene expression experiment; sheet 4: the coleoptile length variation between different alleles of high significant candidate genes.

## Data Availability

The dataset supporting the conclusions of this article is included within the Additional file 10. The targeted resequencing data was previously published in the data descriptor [22] and is publicly available in ArrayExpress under the identifier E-MTAB-7362. The SNP, InDel and KASP genotyping results for the phenology genes are available in Figshare: 10.6084/m9.figshare.c.4357706 [22].

## Abbreviations

GWAS: Genome-wide association studies
SPL3: Squamosa promoter-binding-like protein 3
ZMK1: *Zea mays* K^+^ channel 1
NDP: Nucleoside diphosphate
DH: Doubled haploid
QTL: Quantitative trait locus
MTA: Marker-trait association
CTAB: Cetyl-trimethyl- ammonium bromide
MAF: Minor allele frequency
IBS: Identity-by-state similarity
NJ: Neighbor-Joining
LD: Linkage disequilibrium
PCA: Principal component analysis
CV: Cross validation
GLM: General linear model
Q-Q: Quantile-quantile
FDR: False discovery rate
aa: Amino acid
CSLC6: Cellulose-synthase-like C6
GA2ox: Gibberellin 2-oxidase
GA: Gibberellic acid
EXPB2: Expansin-B2
FT: Flowering Locus T
TPPB: Trehalose-6-phosphate phosphatase
LRR-RLK: Leucine-rich repeat receptor-like protein kinase family
VIN3: Vernalization insensitive 3
BTBD: BTB/POZ domain-containing protein
WA: Western Australia
