# Supplementary material for: Genome-wide association mapping reveals novel genes associated with coleoptile length in a worldwide collection of barley
Source: BMC Plant Biol. 2020 Jul 22;20:346. doi: 10.1186/s12870-020-02547-5 (PMC7374919; doi:10.1186/s12870-020-02547-5)
Supplement: Supplementary file 1 — Additional file 1 Figure S1. Diversity of barley varieties. a) The geographic distribution of barley accessions. Numbers above the bars showed the varieties numbers from each geographical origin. b) Row type and c) growth habit in barley accessions. Numbers on the top of the bars showed the varieties numbers in different row-types and life forms. [file 12870_2020_2547_MOESM1_ESM.docx]

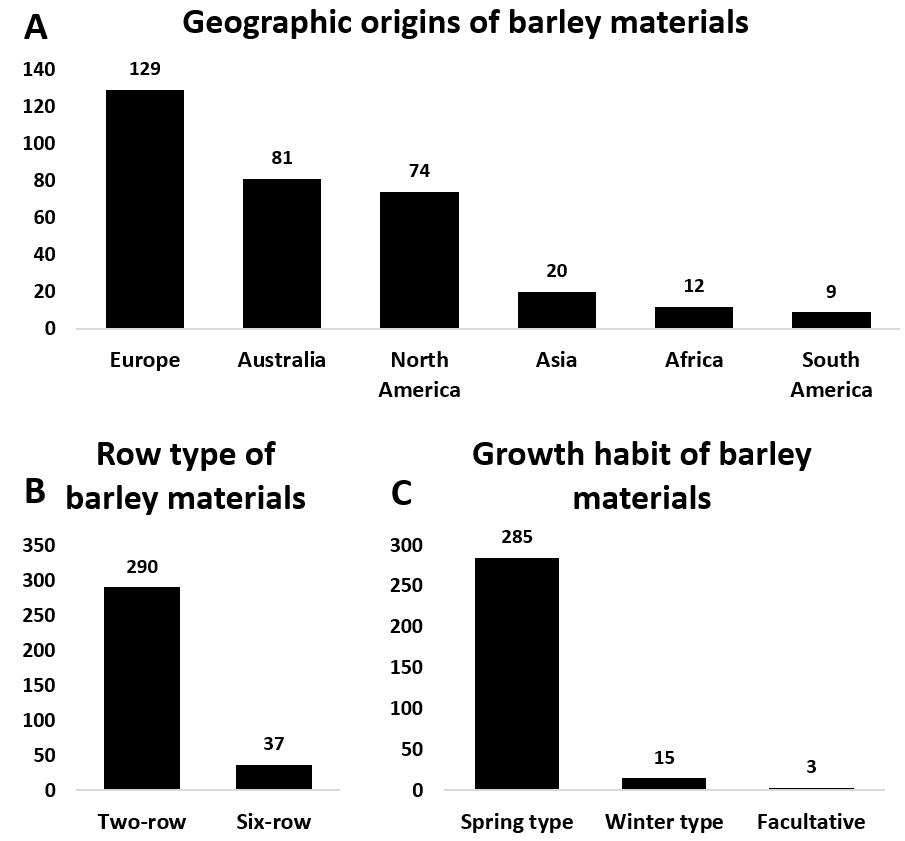


**Figure S1. Diversity of barley varieties.** a) The geographic distribution of barley accessions. Numbers above the bars showed the varieties numbers from each geographical origin. b) Row type and c) growth habit in barley accessions. Numbers on the top of the bars showed the varieties numbers in different row-types and life forms.
