# Supplementary material for: Genome-wide association mapping reveals novel genes associated with coleoptile length in a worldwide collection of barley
Source: BMC Plant Biol. 2020 Jul 22;20:346. doi: 10.1186/s12870-020-02547-5 (PMC7374919; doi:10.1186/s12870-020-02547-5)
Supplement: Supplementary file 2 — Additional file 2 Figure S2. Selection of the optimal number of K (genetic subpopulations). The red line indicated the optimal K value (the most likely number of subpopulations) as 7 based on Δ cross-validation error and standard error in barley accessions. [file 12870_2020_2547_MOESM2_ESM.docx]

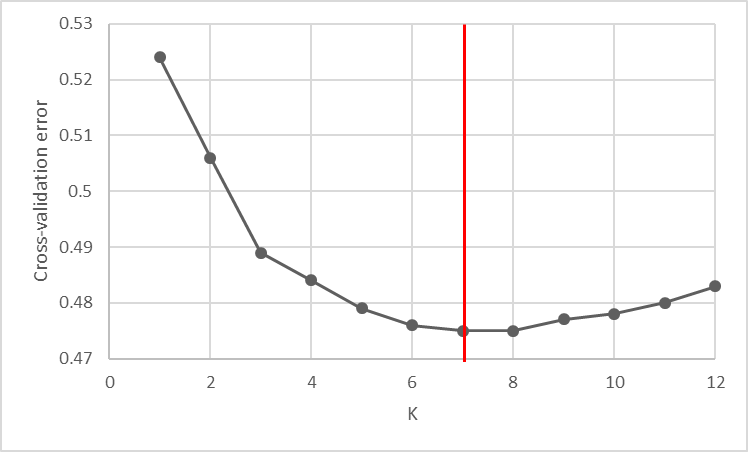


**Figure S2. Selection of the optimal number of K (genetic subpopulations).** The red line indicated the optimal K value (the most likely number of subpopulations) as 7 based on Δ cross-validation error and standard error in barley accessions.
