# Supplementary material for: Genome-wide association mapping reveals novel genes associated with coleoptile length in a worldwide collection of barley
Source: BMC Plant Biol. 2020 Jul 22;20:346. doi: 10.1186/s12870-020-02547-5 (PMC7374919; doi:10.1186/s12870-020-02547-5)
Supplement: Supplementary file 3 — Additional file 3 Figure S3. Population structure analysis using ADMIXTURE for 328 worldwide barley genotypes with 19,014 SNPs. Different populations are showed in different colours. The proportional membership in the population is indicated by the colour of the individual haplotypes. CLUMPP was used to merge the membership coefficients for each population with 100 replicate runs. The number of clusters (K) in 328 barley varieties was determined to be 7 based on the CV error. [file 12870_2020_2547_MOESM3_ESM.docx]

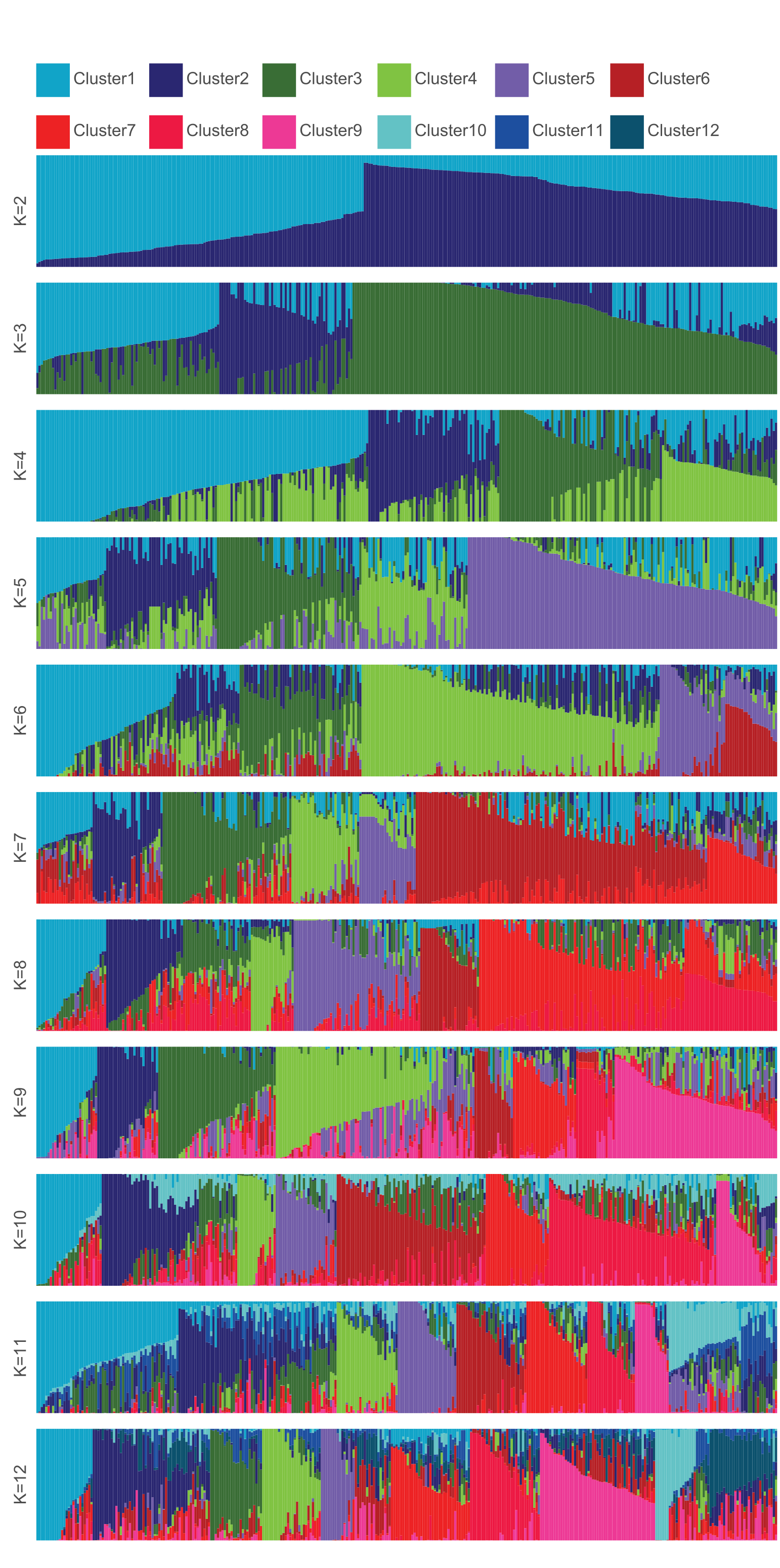


**Figure S3. Population structure analysis using ADMIXTURE for 328 worldwide barley genotypes with 19,014 SNPs.** Different populations are showed in different colours. The proportional membership in the population is indicated by the colour of the individual haplotypes. CLUMPP was used to merge the membership coefficients for each population with 100 replicate runs. The number of clusters (K) in 328 barley varieties was determined to be 7 based on the CV error.
