# Supplementary material for: Genome-wide association mapping reveals novel genes associated with coleoptile length in a worldwide collection of barley
Source: BMC Plant Biol. 2020 Jul 22;20:346. doi: 10.1186/s12870-020-02547-5 (PMC7374919; doi:10.1186/s12870-020-02547-5)
Supplement: Supplementary file 4 — Additional file 4 Figure S4: The LD decay of marker pairs in the worldwide collection of domesticated barley varieties. X-axis indicated the distance (kbp) and Y-axis indicated the mean r2 values of all intra-chromosomal pairs of SNPs. The red lines are the LOESS fitting curves fit by second-degree loess. [file 12870_2020_2547_MOESM4_ESM.docx]

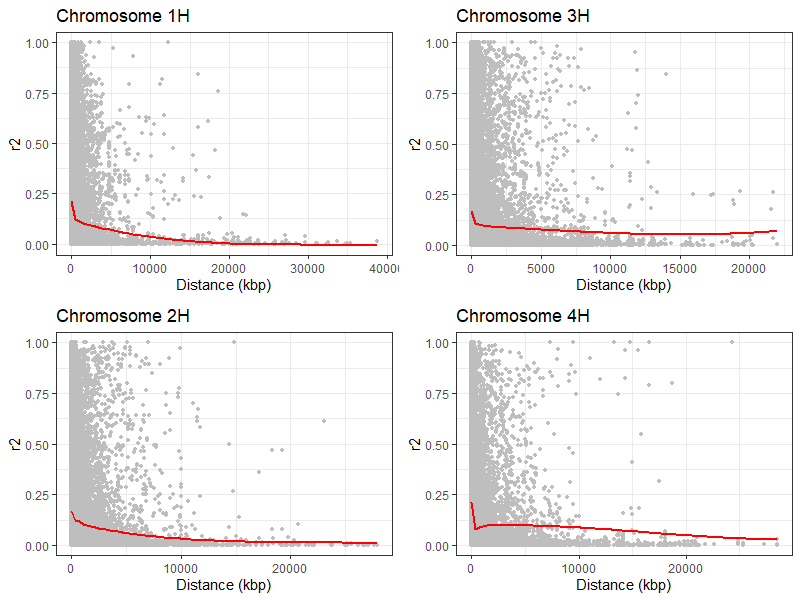


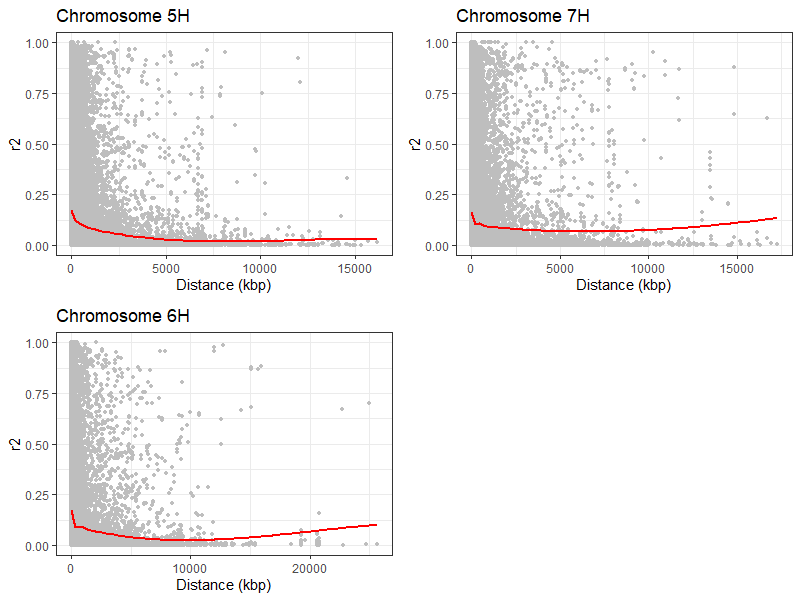


**Figure S4: The LD decay of marker pairs in the worldwide collection of domesticated barley varieties.** X-axis indicated the distance (kbp) and Y-axis indicated the mean r^2^ values of all intra-chromosomal pairs of SNPs. The red lines are the LOESS fitting curves fit by second-degree loess.
