# Supplementary material for: Genome-wide association mapping reveals novel genes associated with coleoptile length in a worldwide collection of barley
Source: BMC Plant Biol. 2020 Jul 22;20:346. doi: 10.1186/s12870-020-02547-5 (PMC7374919; doi:10.1186/s12870-020-02547-5)
Supplement: Supplementary file 5 — Additional file 5 Table S1 Barley accessions with long coleoptile. [file 12870_2020_2547_MOESM5_ESM.docx]

***Table S1 Barley accessions with long coleoptile^†^***

| **Variety** | **Origin** | **Row type** | **Growth habit** | **Coleoptile length (cm)^δ^** |
| --- | --- | --- | --- | --- |
| RUSSIA24 | Europe | two-row | spring | 7.51 |
| 91IBON24 | North America | two-row | spring | 7.46 |
| ESPERANCEORGE289 | n.d.* | two-row | spring | 7.20 |
| CI5791 | Africa | two-row | spring | 7.17 |
| CM67 | North America | six-row | spring | 7.12 |
| EMIRCOMPLEX | Europe | two-row | spring | 6.96 |
| Pearl | Europe | two-row | winter | 6.92 |
| BVDV-026 | North America | two-row | n.d. | 6.91 |
| ND23275 | North America | two-row | n.d. | 6.89 |
| WI4892 | Australia | two-row | spring | 6.89 |
| PISARECKY R 31 | Europe | two-row | spring | 6.87 |
| Hu Mai 4 Hao | Asia | two-row | spring | 6.87 |
| Jyoti-PI 428399 | Asia | six-row | spring | 6.87 |
| Ishuku Shirazu | Asia | two-row | winter | 6.84 |
| Roe | Australia | two-row | spring | 6.75 |
| CI9819 | Africa | two-row | spring | 6.75 |
| Fleet | Australia | two-row | spring | 6.74 |
| Prior | Australia | two-row | spring | 6.70 |
| CHERI | Europe | two-row | spring | 6.68 |
| Maritime | Australia | two-row | spring | 6.63 |
| Apex | Europe | two-row | spring | 6.62 |
| KNEIFLUV P 13 | Europe | two-row | spring | 6.57 |
| RETROARUPOBV-9225 | North America | two-row | spring | 6.56 |
| WI4878 | Australia | two-row | spring | 6.55 |
| Cowabbie | Australia | two-row | spring | 6.52 |
| B585 | Asia | two-row | n.d. | 6.52 |
| WI4859 | Australia | two-row | spring | 6.51 |

^†^ the coleoptile length > 6.50 cm

**^δ^** The coleoptile length was averaged from the sixteen biological and three technical replicates.

* no data
