# Supplementary material for: Genome-wide association mapping reveals novel genes associated with coleoptile length in a worldwide collection of barley
Source: BMC Plant Biol. 2020 Jul 22;20:346. doi: 10.1186/s12870-020-02547-5 (PMC7374919; doi:10.1186/s12870-020-02547-5)
Supplement: Supplementary file 6 — Additional file 6 Table S2 Loci significantly associated with coleoptile length using two MAF (q < 0.05) [file 12870_2020_2547_MOESM6_ESM.docx]

**Table S2 Loci significantly associated with coleoptile length using two MAF (q<0.05)**

| **GLM model MAF>0.05** | | | | | | |
| --- | --- | --- | --- | --- | --- | --- |
| **SNP ID^*^** | **Alleles** | **MAF†** | **R^2^ (%)^§^** | **q value^¶^** | **Candidate gene ID^δ^** | **Annotation** |
| 1H500582726 | C:T | 0.24 | 6.52 | 8.75E-03 | HORVU1Hr1G073010 | unknown function |
| 1H514098702 | A:C | 0.23 | 6.68 | 8.75E-03 | HORVU1Hr1G076430 | Protein FLOWERING LOCUS T |
| 1H516785422 | A:G | 0.20 | 6.28 | 8.75E-03 | HORVU1Hr1G077230 | Cellulose-synthase-like C6 |
| 2H026308852 | A:G | 0.25 | 6.32 | 8.75E-03 | .^ζ^ | . |
| 2H640651652 | G:C | 0.14 | 6.45 | 8.75E-03 | . | . |
| 4H015498974 | C:G | 0.09 | 6.33 | 8.75E-03 | . | . |
| 5H456061421 | A:C | 0.06 | 6.45 | 8.75E-03 | HORVU5Hr1G058300 | trehalose-6-phosphate phosphatase |
| 6H071685909 | C:T | 0.35 | 6.65 | 8.75E-03 | . | . |
| 6H114729800 | T:C | 0.32 | 6.81 | 8.75E-03 | . | . |
| 2H025712787 | C:T | 0.44 | 6.14 | 8.87E-03 | . | . |
| 3H159754241 | A:G | 0.11 | 6.16 | 8.87E-03 | . | . |
| 5H014097066 | A:G | 0.08 | 6.10 | 8.87E-03 | HORVU5Hr1G007340 | Leucine-rich repeat receptor-like protein kinase family protein |
| 6H095840955 | T:C | 0.12 | 6.12 | 8.87E-03 | . | . |
| 1H510799369 | A:C | 0.26 | 6.01 | 9.48E-03 | . | . |
| 3H325190801 | T:G | 0.05 | 6.00 | 9.48E-03 | . | . |
| 4H009849212 | C:T | 0.23 | 5.91 | 9.48E-03 | . | . |
| 6H060392776 | T:C | 0.36 | 5.92 | 9.48E-03 | . | . |
| 6H72969182 | G:A | 0.37 | 5.91 | 9.48E-03 | HORVU6Hr1G022770 | Protein VERNALIZATION INSENSITIVE 3 |
| 6H071745828 | A:G | 0.36 | 5.87 | 9.77E-03 | HORVU6Hr1G022500 | BTB/POZ domain-containing protein |
| 5H596831764 | C:T | 0.10 | 5.78 | 1.05E-02 | HORVU5Hr1G094750 | Protein of unknown function, DUF642 |
| 6H061102980 | A:G | 0.39 | 5.80 | 1.05E-02 | . | . |
| 5H643017198 | T:G | 0.10 | 5.73 | 1.09E-02 | HORVU5Hr1G114030 | receptor kinase 2 |
| 6H071682620 | T:C | 0.32 | 5.72 | 1.09E-02 | . | . |
| 1H516115218 | A:G | 0.26 | 5.65 | 1.12E-02 | HORVU1Hr1G076950 | calcium-transporting ATPase, putative |
| 2H030099714 | C:A | 0.09 | 5.66 | 1.12E-02 | HORVU2Hr1G013900 | Transducin family protein / WD-40 repeat family protein, putative isoform 1 |
| 2H127030114 | G:A | 0.43 | 5.69 | 1.12E-02 | HORVU2Hr1G032570 | SNF1-related protein kinase regulatory subunit gamma-1-like |
| 1H26220293 | T:C | 0.06 | 5.54 | 1.30E-02 | HORVU1Hr1G011030 | COP1-interacting protein-related |
| 3H637358424 | G:A | 0.08 | 5.55 | 1.30E-02 | . | . |
| 3H162295362 | C:T | 0.11 | 5.51 | 1.32E-02 | HORVU3Hr1G032060 | 25.3 kDa vesicle transport protein |
| 4H009062527 | G:A | 0.31 | 5.50 | 1.32E-02 | HORVU4Hr1G004040 | Protein NRT1/ PTR FAMILY 8.3 |
| 6H014496983 | T:C | 0.19 | 5.45 | 1.43E-02 | HORVU6Hr1G007010 | myb domain protein 86 |
| 5H650721872 | G:T | 0.17 | 5.40 | 1.49E-02 | HORVU5Hr1G117440 | Carbohydrate-binding X8 domain superfamily protein |
| 6H058817892 | C:T | 0.36 | 5.40 | 1.49E-02 | . | . |
| 5H428274343 | T:A | 0.10 | 5.38 | 1.49E-02 | . | . |
| 6H071746272 | G:C | 0.05 | 5.36 | 1.53E-02 | HORVU6Hr1G022500 | BTB/POZ domain-containing protein |
| 5H542976088 | G:C | 0.06 | 5.30 | 1.67E-02 | . | . |
| 3H637229883 | T:G | 0.08 | 5.23 | 1.82E-02 | HORVU3Hr1G092020 | BTB/POZ and TAZ domain-containing protein 2 |
| 3H637232108 | C:G | 0.08 | 5.23 | 1.82E-02 | HORVU3Hr1G092020 | BTB/POZ and TAZ domain-containing protein 2 |
| 4H008892994 | C:T | 0.31 | 5.21 | 1.84E-02 | . | . |
| 5H646128430 | A:C | 0.34 | 5.16 | 2.00E-02 | . | . |
| 5H605362844 | C:T | 0.21 | 5.13 | 2.05E-02 | HORVU5Hr1G097710 | Signal peptidase I |
| 6H545054905 | G:C | 0.13 | 5.12 | 2.05E-02 | . | . |
| 2H026103265 | G:T | 0.25 | 5.07 | 2.12E-02 | . | . |
| 2H632188598 | C:G | 0.21 | 5.09 | 2.12E-02 | . | . |
| 4H085418409 | G:T | 0.33 | 5.08 | 2.12E-02 | HORVU4Hr1G018650 | Disease resistance-responsive (dirigent-like protein) family protein |
| 5H665601626 | C:A | 0.26 | 5.03 | 2.24E-02 | HORVU5Hr1G124210 | PGR5-LIKE A |
| 7H585853868 | T:A | 0.05 | 5.03 | 2.24E-02 | . | . |
| 4H014631553 | G:C | 0.11 | 5.01 | 2.29E-02 | HORVU4Hr1G006120 | splicing factor Prp18 family protein |
| 1H514098364 | G:T | 0.21 | 4.98 | 2.32E-02 | HORVU1Hr1G076430 | Protein FLOWERING LOCUS T |
| 7H587772963 | A:G | 0.06 | 4.97 | 2.32E-02 | HORVU7Hr1G096370 | unknown protein |
| 7H636620916 | A:G | 0.11 | 4.98 | 2.32E-02 | HORVU7Hr1G113300 | undescribed protein |
| 2H638375338 | G:A | 0.33 | 4.95 | 2.38E-02 | HORVU2Hr1G089330 | Protein root UVB sensitive 2, chloroplastic |
| 4H06132570 | G:C | 0.09 | 4.93 | 2.40E-02 | . | . |
| 2H127454044 | A:G | 0.42 | 4.90 | 2.44E-02 | . | . |
| 3H039455510 | A:C | 0.34 | 4.91 | 2.44E-02 | HORVU3Hr1G016310 | Pentatricopeptide repeat-containing protein |
| 5H649864513 | T:C | 0.11 | 4.92 | 2.44E-02 | HORVU5Hr1G117000 | Dehydrogenase/reductase SDR family member 4 |
| 6H564705758 | G:C | 0.09 | 4.89 | 2.44E-02 | . | . |
| 2H021376564 | A:G | 0.31 | 4.87 | 2.45E-02 | . | . |
| 3H000585327 | G:A | 0.19 | 4.87 | 2.45E-02 | . | . |
| 6H092522655 | G:A | 0.23 | 4.86 | 2.46E-02 | HORVU6Hr1G025500 | unknown function |
| 3H637569906 | C:T | 0.09 | 4.84 | 2.52E-02 | HORVU3Hr1G092250 | E3 ubiquitin-protein ligase RGLG2 |
| 6H095841002 | C:A | 0.14 | 4.83 | 2.52E-02 | . | . |
| 3H122866902 | G:C | 0.08 | 4.81 | 2.61E-02 | . | . |
| 5H545425272 | C:T | 0.16 | 4.77 | 2.77E-02 | HORVU5Hr1G074910 | Peroxidase superfamily protein |
| 7H652578836 | C:T | 0.10 | 4.76 | 2.77E-02 | . | . |
| 7H652578843 | G:T | 0.10 | 4.76 | 2.77E-02 | . | . |
| 1H513785123 | G:T | 0.24 | 4.75 | 2.79E-02 | HORVU1Hr1G076350 | trehalose-6-phosphate synthase |
| 3H001078120 | T:G | 0.16 | 4.74 | 2.80E-02 | . | . |
| 4H014600422 | A:T | 0.07 | 4.73 | 2.80E-02 | HORVU4Hr1G006090 | unknown function |
| 1H516479984 | G:A | 0.19 | 4.70 | 2.82E-02 | . | . |
| 2H667661509 | C:T | 0.05 | 4.71 | 2.82E-02 | . | . |
| 3H586516867 | T:C | 0.09 | 4.72 | 2.82E-02 | . | . |
| 7H652578833 | C:A | 0.10 | 4.70 | 2.82E-02 | . | . |
| 4H008367264 | T:C | 0.20 | 4.68 | 2.84E-02 | . | . |
| 5H516588248 | G:A | 0.27 | 4.67 | 2.84E-02 | . | . |
| 5H650317758 | C:T | 0.10 | 4.67 | 2.84E-02 | HORVU5Hr1G117080 | phosphate transporter 1;4 |
| 7H652576797 | G:A | 0.09 | 3.86 | 2.84E-02 | . | . |
| 2H036682292 | C:A | 0.33 | 4.65 | 2.92E-02 | . | . |
| 3H588070309 | A:G | 0.05 | 4.63 | 2.97E-02 | . | . |
| 6H061433366 | T:C | 0.35 | 4.63 | 2.97E-02 | . | . |
| 3H637549480 | G:A | 0.07 | 4.60 | 2.98E-02 | . | . |
| 4H005066839 | T:C | 0.17 | 4.61 | 2.98E-02 | HORVU4Hr1G002640 | Pathogenesis-related thaumatin superfamily protein |
| 4H005425389 | G:C | 0.04 | 4.59 | 2.98E-02 | HORVU4Hr1G002870 | Pleckstrin homology (PH) domain-containing protein |
| 7H039911212 | A:G | 0.37 | 4.61 | 2.98E-02 | . | . |
| 7H636588737 | G:A | 0.10 | 4.59 | 2.98E-02 | HORVU7Hr1G113270 | Chitinase family protein |
| UnH249594974 | G:A | 0.12 | 4.61 | 2.98E-02 | HORVU0Hr1G040120 | undescribed protein |
| 4H006536060 | G:A | 0.12 | 4.57 | 3.00E-02 | HORVU4Hr1G003340 | Transcription factor bHLH25 |
| 4H635824203 | C:T | 0.05 | 4.58 | 3.00E-02 | . | . |
| 5H610195095 | T:G | 0.24 | 4.58 | 3.00E-02 | HORVU5Hr1G099410 | Mitochondrial import inner membrane translocase subunit TIM14-1 |
| 2H763607645 | G:A | 0.12 | 4.54 | 3.12E-02 | . | . |
| 7H093418227 | G:T | 0.14 | 4.54 | 3.12E-02 | . | . |
| 2H004038172 | T:G | 0.17 | 4.52 | 3.25E-02 | . | . |
| UnH247874626 | C:A | 0.14 | 4.50 | 3.30E-02 | HORVU0Hr1G039010 | Leucine-rich repeat receptor-like protein kinase family protein |
| 4H639317243 | A:G | 0.28 | 4.48 | 3.40E-02 | . | . |
| 2H028510061 | G:C | 0.20 | 4.47 | 3.41E-02 | HORVU2Hr1G013060 | DOF zinc finger protein 2 |
| 6H022229554 | A:G | 0.17 | 4.48 | 3.41E-02 | . | . |
| 2H738765581 | T:G | 0.16 | 4.45 | 3.42E-02 | . | . |
| 6H014577402 | A:G | 0.19 | 4.45 | 3.42E-02 | HORVU6Hr1G007130 | Chromosome 3B, genomic scaffold, cultivar Chinese Spring |
| 6H015979361 | A:G | 0.28 | 4.46 | 3.42E-02 | . | . |
| 7H026607774 | G:A | 0.29 | 4.46 | 3.42E-02 | HORVU7Hr1G019770 | Eukaryotic aspartyl protease family protein |
| 6H022223662 | T:C | 0.21 | 4.43 | 3.52E-02 | . | . |
| 4H640356258 | T:C | 0.26 | 4.40 | 3.67E-02 | . | . |
| 5H650459008 | G:A | 0.10 | 4.40 | 3.67E-02 | HORVU5Hr1G117110 | lipoxygenase 1 |
| 3H200672079 | C:T | 0.17 | 4.40 | 3.68E-02 | HORVU3Hr1G035840 | Protein kinase superfamily protein |
| 1H500899333 | T:C | 0.16 | 4.39 | 3.70E-02 | . | . |
| 6H38883854 | C:T | 0.04 | 4.38 | 3.75E-02 | . | . |
| 2H738612825 | A:G | 0.18 | 4.34 | 3.94E-02 | HORVU2Hr1G116670 | Cytochrome P450 superfamily protein |
| 3H119256182 | G:A | 0.23 | 4.35 | 3.94E-02 | . | . |
| 7H094105229 | G:A | 0.18 | 4.34 | 3.94E-02 | HORVU7Hr1G038140 | BTB/POZ domain-containing protein |
| 2H713239989 | G:A | 0.05 | 4.33 | 3.96E-02 | . | . |
| 2H764088464 | G:C | 0.43 | 4.32 | 3.96E-02 | . | . |
| 4H007848697 | G:C | 0.12 | 4.32 | 3.96E-02 | HORVU4Hr1G003580 | Phosphomethylpyrimidine synthase |
| 1H509516143 | G:T | 0.24 | 4.29 | 4.09E-02 | . | . |
| 2H026309827 | A:T | 0.33 | 4.30 | 4.09E-02 | . | . |
| 4H008329793 | T:A | 0.09 | 3.50 | 4.09E-02 | . | . |
| 6H112716451 | T:C | 0.05 | 3.49 | 4.09E-02 | . | . |
| 7H620558738 | A:G | 0.28 | 4.28 | 4.09E-02 | . | . |
| 7H637080574 | C:G | 0.15 | 4.28 | 4.09E-02 | HORVU7Hr1G113480 | Acyl-CoA N-acyltransferases (NAT) superfamily protein |
| 7H652578829 | C:G | 0.13 | 4.28 | 4.09E-02 | . | . |
| 1H516264861 | C:G | 0.25 | 4.26 | 4.18E-02 | . | . |
| 1H381530999 | G:A | 0.23 | 4.24 | 4.19E-02 | . | . |
| 1H515520520 | A:G | 0.18 | 4.23 | 4.19E-02 | HORVU1Hr1G076730 | gibberellin 2-oxidase |
| 4H596447653 | T:G | 0.24 | 4.25 | 4.19E-02 | HORVU4Hr1G075070 | PATATIN-like protein 5 |
| 6H015980737 | C:A | 0.25 | 4.24 | 4.19E-02 | . | . |
| 6H015980739 | C:A | 0.25 | 4.24 | 4.19E-02 | . | . |
| 6H095171949 | A:C | 0.14 | 4.24 | 4.19E-02 | HORVU6Hr1G025790 | F-box/RNI-like superfamily protein |
| UnH249594979 | A:C | 0.13 | 4.24 | 4.19E-02 | HORVU0Hr1G040120 | undescribed protein |
| 1H513142352 | C:T | 0.11 | 4.22 | 4.20E-02 | . | . |
| 3H043177202 | A:G | 0.13 | 4.22 | 4.20E-02 | . | . |
| 3H637568987 | A:G | 0.08 | 4.22 | 4.20E-02 | HORVU3Hr1G092250 | E3 ubiquitin-protein ligase RGLG2 |
| 6H460577982 | T:C | 0.37 | 4.22 | 4.20E-02 | HORVU6Hr1G066540 | Protein of unknown function (DUF581) |
| 6H004791252 | C:A | 0.07 | 4.21 | 4.23E-02 | HORVU6Hr1G001490 | translation initiation factor 3 (IF-3) family protein |
| 6H53910792 | T:C | 0.07 | 4.20 | 4.32E-02 | HORVU6Hr1G019700 | Squamosa promoter-binding-like protein 3 |
| 5H668571374 | G:A | 0.12 | 4.16 | 4.57E-02 | . | . |
| 7H624289202 | G:C | 0.10 | 4.16 | 4.57E-02 | HORVU7Hr1G108070 | MATE efflux family protein |
| 3H678757611 | A:G | 0.10 | 4.15 | 4.59E-02 | HORVU3Hr1G109570 | Plant protein of unknown function (DUF247) |
| 4H633601005 | T:C | 0.06 | 4.15 | 4.59E-02 | HORVU4Hr1G086300 | Beta-fructofuranosidase, insoluble isoenzyme 1 |
| 5H444516767 | G:A | 0.10 | 4.15 | 4.59E-02 | . | . |
| 6H563967288 | T:A | 0.11 | 4.14 | 4.59E-02 | . | . |
| 4H221630938 | G:A | 0.17 | 4.11 | 4.86E-02 | . | . |
| 5H415553252 | C:T | 0.16 | 4.11 | 4.86E-02 | . | . |
| 4H000722254 | A:G | 0.13 | 4.10 | 4.88E-02 | HORVU4Hr1G000490 | 2-oxoglutarate (2OG) and Fe(II)-dependent oxygenase superfamily protein |
| 5H643017231 | G:A | 0.14 | 4.10 | 4.92E-02 | HORVU5Hr1G114030 | receptor kinase 2 |
| 6H53911378 | G:A | 0.06 | 4.09 | 4.98E-02 | HORVU6Hr1G019700 | Squamosa promoter-binding-like protein 3 |
| 4H615739094 | T:C | 0.41 | 4.08 | 4.99E-02 | . | . |
| 5H646129817 | G:A | 0.31 | 4.08 | 4.99E-02 | HORVU5Hr1G115280 | Disease resistance-responsive (dirigent-like protein) family protein |
| 7H652582975 | C:T | 0.12 | 4.07 | 4.99E-02 | . | . |
| 1H380633750 | G:A | 0.07 | 4.07 | 5.00E-02 | HORVU1Hr1G051330 | Protein NRT1/ PTR FAMILY 8.3 |
| 1H380634803 | A:G | 0.07 | 4.07 | 5.00E-02 | HORVU1Hr1G051330 | Protein NRT1/ PTR FAMILY 8.3 |
| **GLM model MAF>0.01** | | | | | | |
| **SNP ID** | **Alleles** | **MAF** | **R^2^ (%)** | **q value** | **Candidate gene ID** | **Annotation** |
| 1H514098702 | A:C | 0.23 | 6.71 | 5.45E-03 | HORVU1Hr1G076430 | Protein FLOWERING LOCUS T |
| 4H015498974 | C:G | 0.09 | 6.42 | 5.45E-03 | . | . |
| 6H53910826 | C:T | 0.02 | 5.54 | 5.45E-03 | HORVU6Hr1G019700 | Squamosa promoter-binding-like protein 3 |
| 6H53910924 | T:A | 0.02 | 5.54 | 5.45E-03 | HORVU6Hr1G019700 | Squamosa promoter-binding-like protein 3 |
| 6H53911180 | T:A | 0.02 | 5.54 | 5.45E-03 | HORVU6Hr1G019700 | Squamosa promoter-binding-like protein 3 |
| 6H53911713 | A:C | 0.02 | 5.54 | 5.45E-03 | HORVU6Hr1G019700 | Squamosa promoter-binding-like protein 3 |
| 6H53912147 | C:T | 0.02 | 5.54 | 5.45E-03 | HORVU6Hr1G019700 | Squamosa promoter-binding-like protein 3 |
| 6H53912695 | T:C | 0.02 | 5.54 | 5.45E-03 | HORVU6Hr1G019700 | Squamosa promoter-binding-like protein 3 |
| 6H53913050 | A:C | 0.02 | 5.54 | 5.45E-03 | HORVU6Hr1G019700 | Squamosa promoter-binding-like protein 3 |
| 6H53913075 | C:T | 0.02 | 5.54 | 5.45E-03 | HORVU6Hr1G019700 | Squamosa promoter-binding-like protein 3 |
| 6H53913335 | G:A | 0.02 | 5.54 | 5.45E-03 | HORVU6Hr1G019700 | Squamosa promoter-binding-like protein 3 |
| 6H53913549 | C:T | 0.02 | 5.54 | 5.45E-03 | HORVU6Hr1G019700 | Squamosa promoter-binding-like protein 3 |
| 6H53915124 | T:A | 0.02 | 5.54 | 5.45E-03 | HORVU6Hr1G019700 | Squamosa promoter-binding-like protein 3 |
| 6H071685909 | C:T | 0.35 | 6.53 | 5.45E-03 | . | . |
| 6H114729800 | T:C | 0.32 | 6.69 | 5.45E-03 | . | . |
| 1H500582726 | C:T | 0.24 | 6.37 | 5.50E-03 | HORVU1Hr1G073010 | unknown function |
| 2H640651652 | G:C | 0.14 | 6.35 | 5.50E-03 | . | . |
| 1H516785422 | A:G | 0.20 | 6.29 | 5.63E-03 | HORVU1Hr1G077230 | Cellulose-synthase-like C6 |
| 2H026308852 | A:G | 0.25 | 6.29 | 5.63E-03 | . | . |
| 5H456061421 | A:C | 0.06 | 6.25 | 5.81E-03 | HORVU5Hr1G058300 | trehalose-6-phosphate phosphatase |
| 6H095840955 | T:C | 0.12 | 6.22 | 5.82E-03 | . | . |
| 2H025712787 | C:T | 0.44 | 6.20 | 5.83E-03 | . | . |
| 1H510799369 | A:C | 0.26 | 6.08 | 7.09E-03 | . | . |
| 3H159754241 | A:G | 0.11 | 6.03 | 7.67E-03 | . | . |
| 5H014097066 | A:G | 0.08 | 5.99 | 7.98E-03 | HORVU5Hr1G007340 | Leucine-rich repeat receptor-like protein kinase family protein |
| 3H325190801 | T:G | 0.05 | 5.90 | 9.17E-03 | . | . |
| 4H009849212 | C:T | 0.23 | 5.82 | 1.02E-02 | . | . |
| 6H060392776 | T:C | 0.36 | 5.80 | 1.02E-02 | . | . |
| 6H72969182 | G:A | 0.37 | 5.80 | 1.02E-02 | HORVU6Hr1G022770 | Protein VERNALIZATION INSENSITIVE 3 |
| 1H516115218 | A:G | 0.26 | 5.75 | 1.06E-02 | HORVU1Hr1G076950 | calcium-transporting ATPase, putative |
| 6H071745828 | A:G | 0.36 | 5.76 | 1.06E-02 | HORVU6Hr1G022500 | BTB/POZ domain-containing protein |
| 5H596831764 | C:T | 0.10 | 5.65 | 1.16E-02 | HORVU5Hr1G094750 | Protein of unknown function, DUF642 |
| 5H643017198 | T:G | 0.10 | 5.65 | 1.16E-02 | HORVU5Hr1G114030 | receptor kinase 2 |
| 6H061102980 | A:G | 0.39 | 5.69 | 1.16E-02 | . | . |
| 6H071682620 | T:C | 0.32 | 5.66 | 1.16E-02 | . | . |
| 2H030099714 | C:A | 0.09 | 5.61 | 1.21E-02 | HORVU2Hr1G013900 | Transducin family protein / WD-40 repeat family protein, putative isoform 1 |
| 2H127030114 | G:A | 0.43 | 5.56 | 1.22E-02 | HORVU2Hr1G032570 | SNF1-related protein kinase regulatory subunit gamma-1-like |
| 6H014496983 | T:C | 0.19 | 5.56 | 1.22E-02 | HORVU6Hr1G007010 | myb domain protein 86 |
| 6H53910588 | G:A | 0.02 | 5.55 | 1.22E-02 | HORVU6Hr1G019700 | Squamosa promoter-binding-like protein 3 |
| 6H53915552 | T:C | 0.02 | 5.54 | 1.22E-02 | HORVU6Hr1G019700 | Squamosa promoter-binding-like protein 3 |
| 6H53915657 | G:C | 0.02 | 5.54 | 1.22E-02 | HORVU6Hr1G019700 | Squamosa promoter-binding-like protein 3 |
| 3H637225463 | C:G | 0.05 | 4.65 | 1.31E-02 | HORVU3Hr1G092010 | Chromosome 3B, genomic scaffold, cultivar Chinese Spring |
| 3H637358424 | G:A | 0.08 | 5.45 | 1.42E-02 | . | . |
| 1H26220293 | T:C | 0.06 | 5.42 | 1.47E-02 | HORVU1Hr1G011030 | COP1-interacting protein-related |
| 3H309346397 | G:C | 0.04 | 5.41 | 1.47E-02 | . | . |
| 3H162295362 | C:T | 0.11 | 5.38 | 1.50E-02 | HORVU3Hr1G032060 | 25.3 kDa vesicle transport protein |
| 4H009062527 | G:A | 0.31 | 5.39 | 1.50E-02 | HORVU4Hr1G004040 | Protein NRT1/ PTR FAMILY 8.3 |
| 5H650721872 | G:T | 0.17 | 5.30 | 1.69E-02 | HORVU5Hr1G117440 | Carbohydrate-binding X8 domain superfamily protein |
| 6H058817892 | C:T | 0.36 | 5.30 | 1.69E-02 | . | . |
| 5H542976088 | G:C | 0.06 | 5.27 | 1.75E-02 | . | . |
| 6H071746272 | G:C | 0.05 | 5.25 | 1.80E-02 | HORVU6Hr1G022500 | BTB/POZ domain-containing protein |
| 1H501362076 | G:C | 0.03 | 5.20 | 1.93E-02 | HORVU1Hr1G073150 | O-fucosyltransferase family protein |
| 5H428274343 | T:A | 0.10 | 5.19 | 1.93E-02 | . | . |
| 6H545054905 | G:C | 0.13 | 5.19 | 1.93E-02 | . | . |
| 2H139072555 | T:C | 0.04 | 4.31 | 2.01E-02 | . | . |
| 2H612251760 | C:G | 0.04 | 5.13 | 2.01E-02 | . | . |
| 3H637229883 | T:G | 0.08 | 5.14 | 2.01E-02 | HORVU3Hr1G092020 | BTB/POZ and TAZ domain-containing protein 2 |
| 3H637232108 | C:G | 0.08 | 5.14 | 2.01E-02 | HORVU3Hr1G092020 | BTB/POZ and TAZ domain-containing protein 2 |
| 5H646128430 | A:C | 0.34 | 5.13 | 2.01E-02 | . | . |
| 4H008892994 | C:T | 0.31 | 5.10 | 2.06E-02 | . | . |
| 5H605362844 | C:T | 0.21 | 5.10 | 2.06E-02 | HORVU5Hr1G097710 | Signal peptidase I |
| 1H514098364 | G:T | 0.21 | 5.06 | 2.17E-02 | HORVU1Hr1G076430 | Protein FLOWERING LOCUS T |
| 2H632188598 | C:G | 0.21 | 5.06 | 2.17E-02 | . | . |
| 2H026103265 | G:T | 0.25 | 5.02 | 2.24E-02 | . | . |
| 6H53910349 | T:C | 0.02 | 4.20 | 2.24E-02 | HORVU6Hr1G019700 | Squamosa promoter-binding-like protein 3 |
| 7H616338134 | G:A | 0.04 | 4.19 | 2.24E-02 | HORVU7Hr1G105200 | DNA repair protein RAD51 homolog 4 |
| 4H014631553 | G:C | 0.11 | 4.98 | 2.37E-02 | HORVU4Hr1G006120 | splicing factor Prp18 family protein |
| 4H085418409 | G:T | 0.33 | 4.98 | 2.37E-02 | HORVU4Hr1G018650 | Disease resistance-responsive (dirigent-like protein) family protein |
| 5H597532013 | T:C | 0.04 | 4.93 | 2.59E-02 | HORVU5Hr1G095040 | Beta-glucosidase C |
| 4H06132570 | G:C | 0.09 | 4.92 | 2.63E-02 | . | . |
| 7H636620916 | A:G | 0.11 | 4.90 | 2.68E-02 | HORVU7Hr1G113300 | undescribed protein |
| 1H513785123 | G:T | 0.24 | 4.83 | 2.68E-02 | HORVU1Hr1G076350 | trehalose-6-phosphate synthase |
| 2H021376564 | A:G | 0.31 | 4.84 | 2.68E-02 | . | . |
| 2H638375338 | G:A | 0.33 | 4.85 | 2.68E-02 | HORVU2Hr1G089330 | Protein root UVB sensitive 2, chloroplastic |
| 3H122866902 | G:C | 0.08 | 4.86 | 2.68E-02 | . | . |
| 3H586516867 | T:C | 0.09 | 4.85 | 2.68E-02 | . | . |
| 5H467509665 | T:C | 0.03 | 4.84 | 2.68E-02 | . | . |
| 5H664617845 | G:C | 0.02 | 4.05 | 2.68E-02 | HORVU5Hr1G123670 | Ribonuclease III family protein |
| 5H665601626 | C:A | 0.26 | 4.85 | 2.68E-02 | HORVU5Hr1G124210 | PGR5-LIKE A |
| 6H095841002 | C:A | 0.14 | 4.84 | 2.68E-02 | . | . |
| 6H564705758 | G:C | 0.09 | 4.81 | 2.68E-02 | . | . |
| 7H585853868 | T:A | 0.05 | 4.88 | 2.68E-02 | . | . |
| 7H587772963 | A:G | 0.06 | 4.81 | 2.68E-02 | HORVU7Hr1G096370 | unknown protein |
| 7H652578836 | C:T | 0.10 | 4.81 | 2.68E-02 | . | . |
| 7H652578843 | G:T | 0.10 | 4.81 | 2.68E-02 | . | . |
| UnH114422421 | C:T | 0.04 | 3.99 | 2.68E-02 | HORVU0Hr1G022260 | Chromosome 3B, genomic scaffold, cultivar Chinese Spring |
| 6H092522655 | G:A | 0.23 | 4.80 | 2.68E-02 | HORVU6Hr1G025500 | unknown function |
| 1H516479984 | G:A | 0.19 | 4.77 | 2.72E-02 | . | . |
| 2H127454044 | A:G | 0.42 | 4.77 | 2.72E-02 | . | . |
| 5H649864513 | T:C | 0.11 | 4.77 | 2.72E-02 | HORVU5Hr1G117000 | Dehydrogenase/reductase SDR family member 4 |
| 7H652576797 | G:A | 0.09 | 3.97 | 2.72E-02 | . | . |
| 3H000585327 | G:A | 0.19 | 4.75 | 2.80E-02 | . | . |
| 7H652578833 | C:A | 0.10 | 4.75 | 2.80E-02 | . | . |
| 4H630956532 | C:T | 0.04 | 4.73 | 2.84E-02 | HORVU4Hr1G085140 | Remorin family protein |
| 3H039455510 | A:C | 0.34 | 4.71 | 2.86E-02 | HORVU3Hr1G016310 | Pentatricopeptide repeat-containing protein |
| 3H637569906 | C:T | 0.09 | 4.71 | 2.86E-02 | HORVU3Hr1G092250 | E3 ubiquitin-protein ligase RGLG2 |
| 5H598560301 | T:C | 0.04 | 4.71 | 2.86E-02 | HORVU5Hr1G095530 | phytochrome C |
| 5H601590546 | G:A | 0.04 | 4.72 | 2.86E-02 | HORVU5Hr1G096560 | Disease resistance protein |
| 5H545425272 | C:T | 0.16 | 4.70 | 2.90E-02 | HORVU5Hr1G074910 | Peroxidase superfamily protein |
| 3H588070309 | A:G | 0.05 | 4.67 | 3.02E-02 | . | . |
| 1H517127039 | G:A | 0.03 | 3.85 | 3.05E-02 | . | . |
| 2H706099652 | C:G | 0.02 | 3.85 | 3.05E-02 | HORVU2Hr1G105240 | purine permease 11 |
| 4H635824203 | C:T | 0.05 | 4.65 | 3.07E-02 | . | . |
| 4H008367264 | T:C | 0.20 | 4.64 | 3.09E-02 | . | . |
| 3H001078120 | T:G | 0.16 | 4.64 | 3.09E-02 | . | . |
| 5H666428715 | C:A | 0.03 | 4.63 | 3.11E-02 | HORVU5Hr1G124450 | Heavy metal transport/detoxification superfamily protein |
| 4H014600422 | A:T | 0.07 | 4.62 | 3.14E-02 | HORVU4Hr1G006090 | unknown function |
| 2H012663546 | T:C | 0.02 | 3.78 | 3.19E-02 | . | . |
| 2H012690993 | T:C | 0.02 | 3.78 | 3.19E-02 | . | . |
| 2H036682292 | C:A | 0.33 | 4.60 | 3.19E-02 | . | . |
| 2H763607645 | G:A | 0.12 | 4.58 | 3.19E-02 | . | . |
| 6H015979361 | A:G | 0.28 | 4.60 | 3.19E-02 | . | . |
| 6H156668471 | C:A | 0.02 | 4.59 | 3.19E-02 | . | . |
| 7H093418227 | G:T | 0.14 | 4.58 | 3.19E-02 | . | . |
| 4H006536060 | G:A | 0.12 | 4.58 | 3.19E-02 | HORVU4Hr1G003340 | Transcription factor bHLH25 |
| UnH249594974 | G:A | 0.12 | 4.57 | 3.19E-02 | HORVU0Hr1G040120 | undescribed protein |
| 2H667661509 | C:T | 0.05 | 4.55 | 3.24E-02 | . | . |
| 4H005066839 | T:C | 0.17 | 4.55 | 3.24E-02 | HORVU4Hr1G002640 | Pathogenesis-related thaumatin superfamily protein |
| 5H516588248 | G:A | 0.27 | 4.55 | 3.24E-02 | . | . |
| 6H014577402 | A:G | 0.19 | 4.56 | 3.24E-02 | HORVU6Hr1G007130 | Chromosome 3B, genomic scaffold, cultivar Chinese Spring |
| 6H061433366 | T:C | 0.35 | 4.55 | 3.24E-02 | . | . |
| 5H666428377 | G:C | 0.03 | 4.54 | 3.28E-02 | HORVU5Hr1G124450 | Heavy metal transport/detoxification superfamily protein |
| 5H650317758 | C:T | 0.10 | 4.53 | 3.30E-02 | HORVU5Hr1G117080 | phosphate transporter 1;4 |
| 7H636588737 | G:A | 0.10 | 4.52 | 3.34E-02 | HORVU7Hr1G113270 | Chitinase family protein |
| 2H019328009 | A:G | 0.02 | 4.51 | 3.38E-02 | . | . |
| 3H637549480 | G:A | 0.07 | 4.49 | 3.46E-02 | . | . |
| 6H38883854 | C:T | 0.04 | 4.49 | 3.46E-02 | . | . |
| 7H039911212 | A:G | 0.37 | 4.49 | 3.47E-02 | . | . |
| 2H028510061 | G:C | 0.20 | 4.47 | 3.47E-02 | HORVU2Hr1G013060 | DOF zinc finger protein 2 |
| 2H738765581 | T:G | 0.16 | 4.47 | 3.47E-02 | . | . |
| 4H005425389 | G:C | 0.04 | 4.48 | 3.47E-02 | HORVU4Hr1G002870 | Pleckstrin homology (PH) domain-containing protein |
| 4H639317243 | A:G | 0.28 | 4.47 | 3.47E-02 | . | . |
| 5H610195095 | T:G | 0.24 | 4.47 | 3.47E-02 | HORVU5Hr1G099410 | Mitochondrial import inner membrane translocase subunit TIM14-1 |
| 1H398310882 | G:C | 0.05 | 4.43 | 3.64E-02 | HORVU1Hr1G053950 | unknown protein |
| 2H004038172 | T:G | 0.17 | 4.43 | 3.64E-02 | . | . |
| 6H022223662 | T:C | 0.21 | 4.43 | 3.64E-02 | . | . |
| 2H764088464 | G:C | 0.43 | 4.43 | 3.65E-02 | . | . |
| 3H200672079 | C:T | 0.17 | 4.42 | 3.67E-02 | HORVU3Hr1G035840 | Protein kinase superfamily protein |
| 6H562139666 | T:A | 0.03 | 4.41 | 3.69E-02 | . | . |
| 6H022229554 | A:G | 0.17 | 4.41 | 3.71E-02 | . | . |
| 3H119256182 | G:A | 0.23 | 4.39 | 3.84E-02 | . | . |
| UnH247874626 | C:A | 0.14 | 4.38 | 3.85E-02 | HORVU0Hr1G039010 | Leucine-rich repeat receptor-like protein kinase family protein |
| 4H640356258 | T:C | 0.26 | 4.38 | 3.88E-02 | . | . |
| 2H713239989 | G:A | 0.05 | 4.37 | 3.90E-02 | . | . |
| 1H509516143 | G:T | 0.24 | 4.35 | 3.96E-02 | . | . |
| 6H015980737 | C:A | 0.25 | 4.35 | 3.96E-02 | . | . |
| 6H015980739 | C:A | 0.25 | 4.35 | 3.96E-02 | . | . |
| 1H500899333 | T:C | 0.16 | 4.34 | 3.99E-02 | . | . |
| 2H738612825 | A:G | 0.18 | 4.34 | 3.99E-02 | HORVU2Hr1G116670 | Cytochrome P450 superfamily protein |
| 3H637080819 | C:G | 0.04 | 3.54 | 3.99E-02 | . | . |
| 4H007848697 | G:C | 0.12 | 4.34 | 3.99E-02 | HORVU4Hr1G003580 | Phosphomethylpyrimidine synthase |
| 1H516264861 | C:G | 0.25 | 4.33 | 4.02E-02 | . | . |
| 7H094105229 | G:A | 0.18 | 4.33 | 4.02E-02 | HORVU7Hr1G038140 | BTB/POZ domain-containing protein |
| 7H026607774 | G:A | 0.29 | 4.31 | 4.10E-02 | HORVU7Hr1G019770 | Eukaryotic aspartyl protease family protein |
| 1H515520520 | A:G | 0.18 | 4.31 | 4.11E-02 | HORVU1Hr1G076730 | gibberellin 2-oxidase |
| 1H400987989 | C:G | 0.03 | 3.49 | 4.28E-02 | HORVU1Hr1G054230 | expansin B2 |
| 5H650459008 | G:A | 0.10 | 4.28 | 4.29E-02 | HORVU5Hr1G117110 | lipoxygenase 1 |
| 7H652578829 | C:G | 0.13 | 4.27 | 4.32E-02 | . | . |
| 2H026309827 | A:T | 0.33 | 4.26 | 4.44E-02 | . | . |
| 3H350514395 | G:C | 0.03 | 3.46 | 4.45E-02 | HORVU3Hr1G049640 | calmodulin-binding family protein |
| 1H513142352 | C:T | 0.11 | 4.25 | 4.48E-02 | . | . |
| 6H53915829 | G:A | 0.02 | 4.24 | 4.48E-02 | HORVU6Hr1G019700 | Squamosa promoter-binding-like protein 3 |
| 6H095171949 | A:C | 0.14 | 4.24 | 4.48E-02 | HORVU6Hr1G025790 | F-box/RNI-like superfamily protein |
| 3H076045034 | G:C | 0.04 | 3.44 | 4.51E-02 | HORVU3Hr1G021970 | Transcription initiation factor IIA subunit 2 |
| 5H663398403 | C:A | 0.02 | 3.44 | 4.51E-02 | HORVU5Hr1G123160 | DERLIN-2.2 |
| 7H637080574 | C:G | 0.15 | 4.23 | 4.54E-02 | HORVU7Hr1G113480 | Acyl-CoA N-acyltransferases (NAT) superfamily protein |
| UnH249594979 | A:C | 0.13 | 4.21 | 4.64E-02 | HORVU0Hr1G040120 | undescribed protein |
| UnH249595059 | G:A | 0.02 | 4.21 | 4.64E-02 | HORVU0Hr1G040120 | undescribed protein |
| 2H701643794 | C:T | 0.04 | 4.20 | 4.72E-02 | HORVU2Hr1G104030 | Receptor-like protein kinase 2 |
| 7H616338325 | C:T | 0.03 | 4.19 | 4.74E-02 | HORVU7Hr1G105200 | DNA repair protein RAD51 homolog 4 |
| 6H72973238 | G:A | 0.04 | 3.39 | 4.82E-02 | HORVU6Hr1G022770 | Protein VERNALIZATION INSENSITIVE 3 |
| 7H652582975 | C:T | 0.12 | 4.18 | 4.83E-02 | . | . |
| 2H692438251 | G:A | 0.02 | 3.37 | 4.85E-02 | HORVU2Hr1G100970 | 60S ribosomal protein L7-4 |
| 4H008329793 | T:A | 0.09 | 3.38 | 4.85E-02 | . | . |
| 6H004791252 | C:A | 0.07 | 4.16 | 4.85E-02 | HORVU6Hr1G001490 | translation initiation factor 3 (IF-3) family protein |
| 6H460577982 | T:C | 0.37 | 4.17 | 4.85E-02 | HORVU6Hr1G066540 | Protein of unknown function (DUF581) |
| 7H620558738 | A:G | 0.28 | 4.17 | 4.85E-02 | . | . |

*the ID consists of the chromosome number followed by the marker’s physical position

†minor allele frequency

^§^contribution to phenotypic variation

^¶^adjusted P value, significant at q<0.05; the association list of q< 0.01 see table 2

^δ^annotated in barley genome assembly IBSC v

^ζ^ intergenic region
