# Supplementary material for: Genome-wide association mapping reveals novel genes associated with coleoptile length in a worldwide collection of barley
Source: BMC Plant Biol. 2020 Jul 22;20:346. doi: 10.1186/s12870-020-02547-5 (PMC7374919; doi:10.1186/s12870-020-02547-5)
Supplement: Supplementary file 7 — Additional file 7 Table S3 Intergenic Loci with highly significant association (qFDR < 0.01) and candidate genes [file 12870_2020_2547_MOESM7_ESM.docx]

**Table S3 Intergenic Loci with highly significant association (qFDR<0.01) and candidate genes**

| **Marker name** | **Chromosome** | **Position** | **Candidate gene** | **Gene ID*** | **Distance (Mb)** † |
| --- | --- | --- | --- | --- | --- |
| D2H026308852 | 2H | 26,308,852 | not found | not found | not found |
| D2H640651652 | 2H | 640,651,652 | *auxin response factor 10* | *HORVU2Hr1G089660* | 0.42 |
|  |  |  | *Gibberellin 2-beta-dioxygenase 8* | *HORVU2Hr1G090030* | 1.22 |
| D4H015498974 | 4H | 15,498,974 | not found | not found | not found |
| D6H071685909 | 6H | 71,685,909 | *Endoglucanase 14* | *HORVU6Hr1G022400* | 0.76 |
| D6H114729800 | 6H | 114,729,800 | not found | not found | not found |
| D2H025712787 | 2H | 25,712,787 | not found | not found | not found |
| D3H159754241 | 3H | 159,754,241 | *elongation defective 1 protein / ELD1 protein* | *HORVU3Hr1G031800* | 0.16 |
| D6H095840955 | 6H | 95,840,955 | not found | not found | not found |
| D1H510799369 | 1H | 510,799,369 | not found | not found | not found |
| L3H325190801 | 3H | 325,190,801 | not found | not found | not found |
| D4H009849212 | 4H | 9,849,212 | *H(+)-ATPase 11* | *HORVU4Hr1G004820* | 0.71 |
| D6H060392776 | 6H | 60,392,776 | *SAUR-like auxin-responsive protein family* | *HORVU6Hr1G020840* | 0.09 |

***** annotated in barley genome assembly IBSC v2

†physical distance from the marker to the candidate gene
