# Supplementary material for: Genome-wide association mapping reveals novel genes associated with coleoptile length in a worldwide collection of barley
Source: BMC Plant Biol. 2020 Jul 22;20:346. doi: 10.1186/s12870-020-02547-5 (PMC7374919; doi:10.1186/s12870-020-02547-5)
Supplement: Supplementary file 8 — Additional file 8 Figre S5. Manhattan plots and quantile–quantile (QQ) plot of coleoptile length. (A) The Manhattan plot of coleoptile length by GLM model using minor allele frequency (MAF) < 0.05. (B) The Manhattan plot of coleoptile length by GLM model using minor allele frequency (MAF) < 0.01. The significance of marker-trait associations was presented using q values (−log10 of FDR adjusted p-values). The blue dashed lines indicated the significant threshold at qFDR< 0.05 and the red dashed lines indicated the significant threshold at qFDR< 0.01. (C) QQ plot for coleoptile length using q values (−log10 of FDR adjusted p-values) by GLM model with MAF < 0.05. (D) QQ plot for coleoptile length using q values by GLM model with MAF < 0.01. [file 12870_2020_2547_MOESM8_ESM.docx]

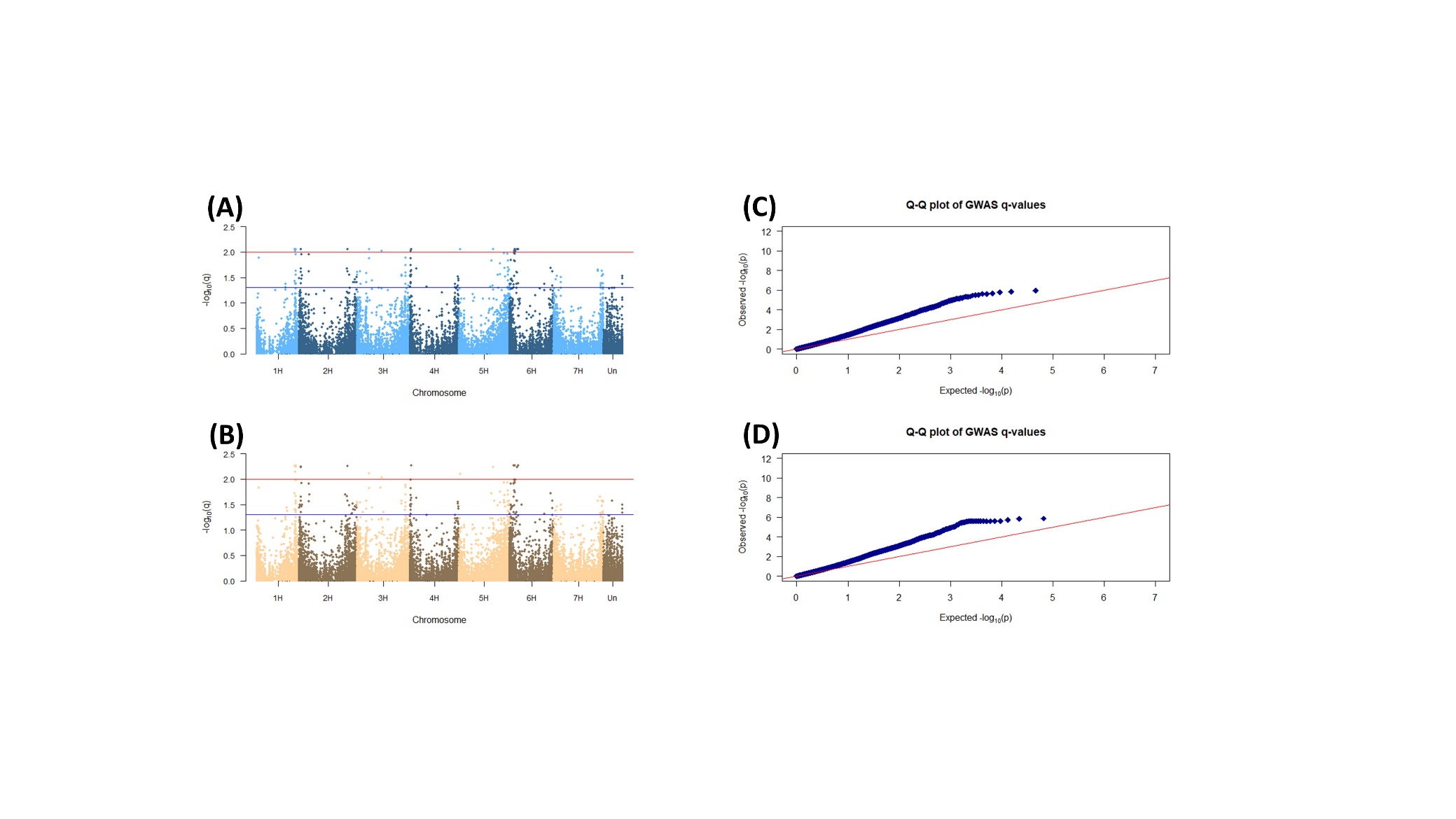


**Figure S5. Manhattan plots and quantile–quantile (QQ) plot of coleoptile length.** (A) The Manhattan plot of coleoptile length by GLM model using minor allele frequency (MAF)<0.05. (B) The Manhattan plot of coleoptile length by GLM model using minor allele frequency (MAF)<0.01. The significance of marker-trait associations was presented using q values (-log10 of FDR adjusted p-values). The blue dashed lines indicated the significant threshold at qFDR<0.05 and the red dashed lines indicated the significant threshold at qFDR<0.01. (C) QQ plot for coleoptile length using q values (-log10 of FDR adjusted p-values) by GLM model with MAF<0.05. (D) QQ plot for coleoptile length using q values by GLM model with MAF<0.01.
