# Supplementary material for: Genome-wide association mapping reveals novel genes associated with coleoptile length in a worldwide collection of barley
Source: BMC Plant Biol. 2020 Jul 22;20:346. doi: 10.1186/s12870-020-02547-5 (PMC7374919; doi:10.1186/s12870-020-02547-5)
Supplement: Supplementary file 9 — Additional file 9 Figure S6. The amino acid substitution site and the conserved motifs on SBP domain of gene SPL3. Zn-1: zinc finger 1; Zn-2: zinc finger 2; NLS: nuclear localization signal. Blue colour indicated the most conserved region on zinc finger 1. Red colour indicated the most conserved region on zinc finger 2. Purple colour indicated the most conserved region on nuclear localization signal. [file 12870_2020_2547_MOESM9_ESM.docx]

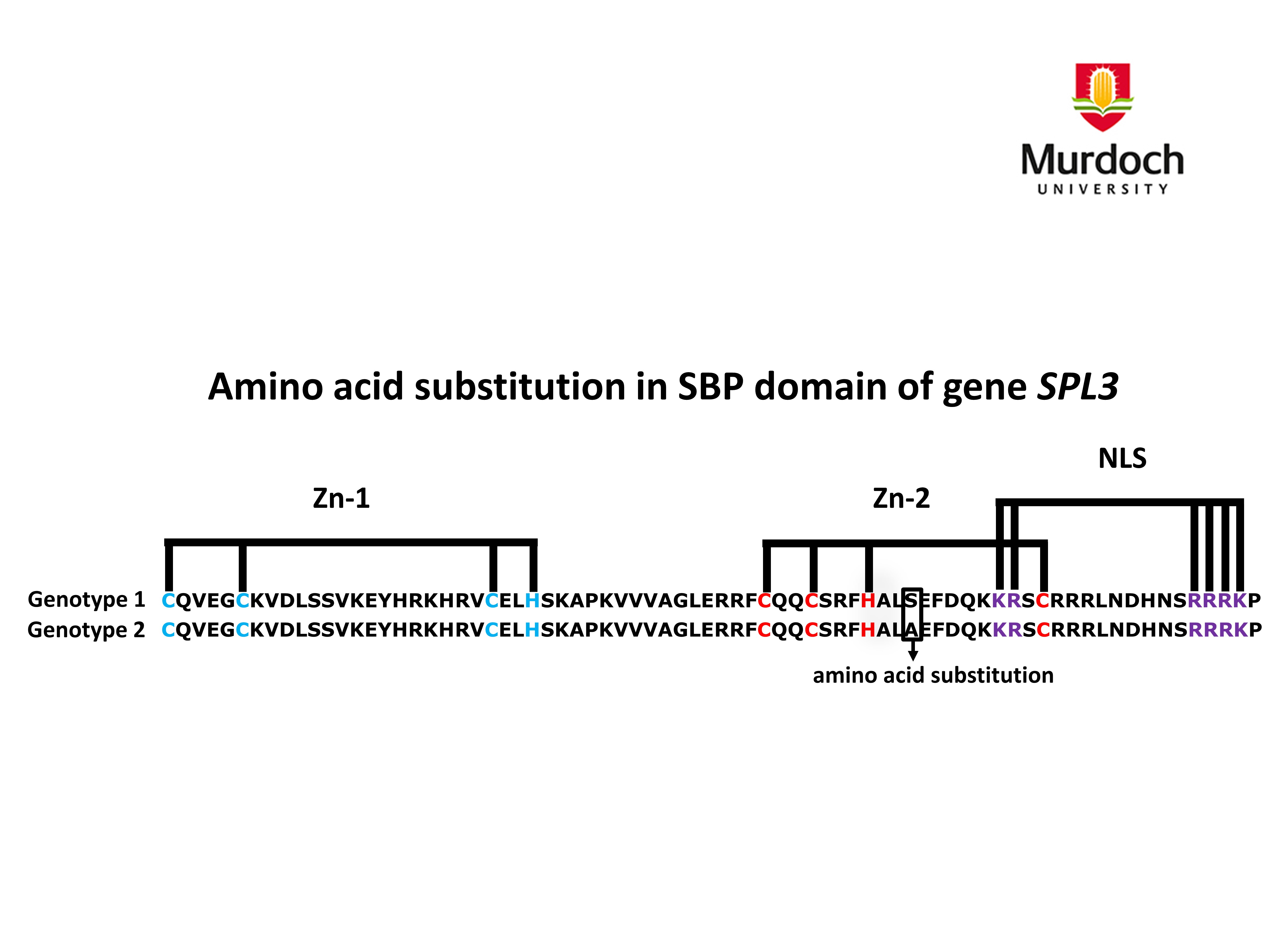


**Figure S6.** The amino acid substitution site and the conserved motifs on SBP domain of gene *SPL3*. Zn-1: zinc finger 1; Zn-2: zinc finger 2; NLS: nuclear localization signal. Blue colour indicated the most conserved region on zinc finger 1. Red colour indicated the most conserved region on zinc finger 2. Purple colour indicated the most conserved region on nuclear localization signal.
